# Effects of fallow tillage on winter wheat yield and predictions under different precipitation types

**DOI:** 10.7717/peerj.12602

**Published:** 2021-12-08

**Authors:** Yu Feng, Wen Lin, Shaobo Yu, Aixia Ren, Qiang Wang, Hafeez Noor, Jianfu Xue, Zhenping Yang, Min Sun, Zhiqiang Gao

**Affiliations:** Shanxi Agricultural University, Taiyuan, Shanxi, China; State Key Laboratory of Sustainable Dryland Agriculture (In preparation), Shanxi Agricultural University, Taiyuan, Shanxi, China

**Keywords:** Precipitation types, Random Forest, Tillage, Winter wheat, Yield prediction

## Abstract

In northern China, precipitation that is primarily concentrated during the fallow period is insufficient for the growth stage, creates a moisture shortage, and leads to low, unstable yields. Yield prediction in the early growth stages significantly informs field management decisions for winter wheat (*Triticum aestivum* L.). A 10-year field experiment carried out in the Loess Plateau area tested how three tillage practices (deep ploughing (DP), subsoiling (SS), and no tillage (NT)) influenced cultivation and yield across different fallow periods. The experiment used the random forest (RF) algorithm to construct a prediction model of yields and yield components. Our results revealed that tillage during the fallow period was more effective than NT in improving yield in dryland wheat. Under drought condition, DP during the fallow period achieved a higher yield than SS, especially in drought years; DP was 16% higher than SS. RF was deemed fit for yield prediction across different precipitation years. An RF model was developed using meteorological factors for fixed variables and soil water storage after tillage during a fallow period for a control variable. Small error values existed in the prediction yield, spike number, and grains number per spike. Additionally, the relative error of crop yield under fallow tillage (5.24%) was smaller than that of NT (6.49%). The prediction error of relative meteorological yield was minimum and optimal, indicating that the model is suitable to explain the influence of meteorological factors on yield.

## Introduction

Approximately 80% of rain-fed farmland provides approximately 60% of the global food supply. Rain-fed agriculture accounts for approximately 70% of Asian and Pacific regions’ arable lands (FAO, 2019). The Loess Plateau is the primary wheat-producing region in China, and dryland farmlands account for 80% of the cultivated land in the region, which has great potential for increasing production (Li et al., 2009; Zhang et al., 2009). The predominant cropping pattern of dryland wheat traditionally includes annual cultivation, *i.e*., a drylad winter wheat (*Triticum aestivum* L.)-summer fallow system. Given the constraints presented by insufficient rainfall, large interannual variability, low soil fertility, and strong evapotranspiration, this region’s yield of dryland winter wheat has been substantially lower than the average yield in other areas of China and European countries (Qiu, Hao & Wu, 2017; Jia et al., 2020; Du et al., 2020). Improving dryland wheat yield and comprehensive production technology has been the frequent focus of recent research.

Precipitation during the fallow period in China greatly affects soil moisture accumulation during the rain-fed agricultural growth stage, ultimately influencing yield contingent upon the efficiencies of nutrient and water utilization (Li et al., 2019). Consequently, the water required by dryland winter wheat growth is the most important limiting factor for yield improvement. Deep ploughing (DP) and subsoiling (SS) are common fallow tillage methods used in northern China, and there are many studies on improving soil water use efficiency and yield. A 5-year field experiment found that compared to no tillage (NT), DP and SS effectively increased soil water storage to 0–3 m, increased soil porosity and significantly improved water use efficiency (WUE) and yield (Sun et al., 2018). The dryland winter wheat yield under different fallow tillage methods was in direct proportion to the levels of precipitation during the fallow period (Xue et al., 2019). Using tillage during the fallow period can meet water demands during the growth period. This is conducive to the formation of the number of spikes and grains number per spike, which increases the yield (Ren et al., 2019). In the Loess Plateau, it was shown that DP increased soil water storage during the sowing stage, and significantly improved yield, 1,000-grains weight, WUE, and nitrogen use efficiency (Khan et al., 2020). SS increased soil porosity, increased soil moisture in the cultivated layer, and improved WUE, thereby promoting crop growth and increasing yield (Shen et al., 2021).

The Loess Plateau is located in a monsoon climate zone at the middle latitudes (Mo et al., 2016). Under current climate change conditions where variability in annual precipitation has increased, there are often large differences in precipitation levels during the fallow period across different years, which affects the accumulation of soil moisture and leads to different yields of dryland winter wheat at harvest (IPCC, 2014). The previous classification of hydrological year type was mostly based on annual precipitation. The effects of different tillage methods on crop water consumption and yield has been discussed, but there have been few studies on yield prediction (Yu et al., 2020). The Standardized Precipitation Evapotranspiration Index (SPEI), one of the most commonly-used agricultural drought indices, not only considers precipitation, but also the impact of temperature on crop growth (Feng et al., 2019; Shaukat et al., 2020).

Yield prediction during the early growth stages of dryland winter wheat is of great significance for the formulation of field management measures. Developments in computer technology and machine learning methods have also been applied in crop yield prediction research in recent years and have achieved good results (Basha et al., 2020). The random forest (RF) algorithm is a model that has performed better than other methods in crop yield prediction, and has a satisfactory tolerance to outliers and noise. This model has been widely used in medicine, agronomy, biology, atmospheric science, and other fields (Tulbure et al., 2012; Banerjee et al., 2015; Rubal & Kumar, 2018). The RF algorithm has been used to generate prediction models for sugarcane and has become one of the seasonal yield prediction models used for complex crops in Canada (Newlands et al., 2014; Everingham et al., 2016). Compared to different models used in the prediction of soybean production genomes, the RF model has shown widely adaptive potential (Đorđević et al., 2019). RF had the best performance when predicting maize yield after comparing four machine algorithms (Ramos et al., 2020). Although previous studies using RF have achieved reliable prediction effects, predictive research on the yield of grain crops and components has been insufficient.

In this study, we classified the types of cultivated wheat across different years based on SPEI during the fallow period. We then analyzed the effects of yield and fallow tillage methods in different years with the purpose of identifying suitable fallow tillage methods on different types to obtain the highest yields. In addition to using the main meteorological factors affecting wheat growth during the early stage as modelling factors, soil water storage factors caused by changes in tillage methods were also used in the construction of the RF model for the early prediction of winter wheat yield and its components. Ultimately, the objectives of this research were to: (1) classify wheat cultivation year types using the SPEI, (2) analyze the impact of fallow tillage on winter wheat yield and components across different year types, (3) construct the RF model using meteorological factors and soil water storage, and (4) predict and assess yield and components under different tillage methods.

## Materials & Methods

### Research site description

A 10-year field experiment was conducted at the Wenxi Dryland Wheat Agriculture Station (111°17′E, 35°20′N), Shanxi Province, China, between 2009 and 2018. The experiment was performed on a winter wheat-summer fallow system, which is land that is bare and unsowned from the harvest of the previous crop to the next wheat crop. The site is characterized by the typical warm-temperate, semi-drought, continental monsoon climate of the south-eastern Loess Plateau, with an average annual temperature of 13.72 °C, 2,461 h of annual sunshine, 1,838.9 mm of annual evaporation from the free water surface, and 491 mm of average annual precipitation (Table 1). The experimental field is hilly dryland with no irrigation, yellow-thorn soil (A11-Btk-Bk type according to Chinese soil taxonomy), a deep soil layer, significant profile differentiation, a pH of 8.0–8.3, good permeability, and medium fertility. The nutrient content and geomorphologic characteristics of the experiment site are representative of the Loess Plateau’s gully region (Table 2).

**Table 1:** Precipitation distribution during both fallow and growing seasons. Precipitation during the study (2009–2018) and the difference in average precipitation across the last 35 years (1981–2017) in different growth stages of wheat at the experimental site in Wenxi, China.

	2009	2010	2011	2012	2013	2014	2015	2016	2017	2018	1981–2017 Average
Fallow Stage	206.2	390.0	506.8	218.9	210.7	472.4	214.8	184.2	196.9	234.4	284.6
Sowing-Jointing Stage	82.2	75.6	165.6	52.4	62.9	100.1	58.3	196.9	125.1	46.5	88.6
Jointing-Anthesis Stage	28.5	24.3	15.5	25.1	104.7	38.4	40.9	26.0	84.7	36.7	37.6
Anthesis-Maturity Stage	65.0	27.1	45.1	95.2	35.9	29.3	76.8	77.4	50.1	41.4	80.2
Whole growth period	175.7	127.0	226.2	172.7	203.5	167.8	176.0	300.3	259.9	124.6	206.4
Total	381.9	517.0	733.0	391.6	414.2	640.2	390.8	484.5	456.8	359.0	491.0

**Note:**

The data were from meteorological observation station of Wenxi County, Shanxi Province, China. Fallow stage was from the last 10 d of June to the last 10 d of September, before Sowing-Jointing stage was from the first 10 d of October to the first 10 d of April in the following year, Jointing-Anthesis stage was from the middle 10 d of April to the first 10 d of May, Anthesis-Maturity stage was from the middle 10 d of May to the middle 10 d of June.

**Table 2:** Soil nutrient properties from the experimental location in Shanxi (2009–2016). We analyzed 0–0.20 m of soil at the research site (2009–2016) for its including organic matter, total nitrogen, alkaline hydrolysis nitrogen, and available phosphorus before sowing.

Year	Organic matter (g·kg^−1^)	Total nitrogen (g·kg^−1^)	Alkaline hydrolysis nitrogen (mg·kg^−1^)	Available phosphorus (mg·kg^−1^)
2009	10.65	0.74	32.93	20.08
2010	10.75	0.72	32.91	19.89
2011	10.72	0.78	40.16	19.87
2012	10.38	0.71	38.62	16.61
2013	10.18	0.70	39.32	16.62
2014	10.55	0.68	37.65	17.64
2015	11.16	0.67	32.79	15.67
2016	10.62	0.69	38.22	15.28

### Experimental design

**Figure 1:**
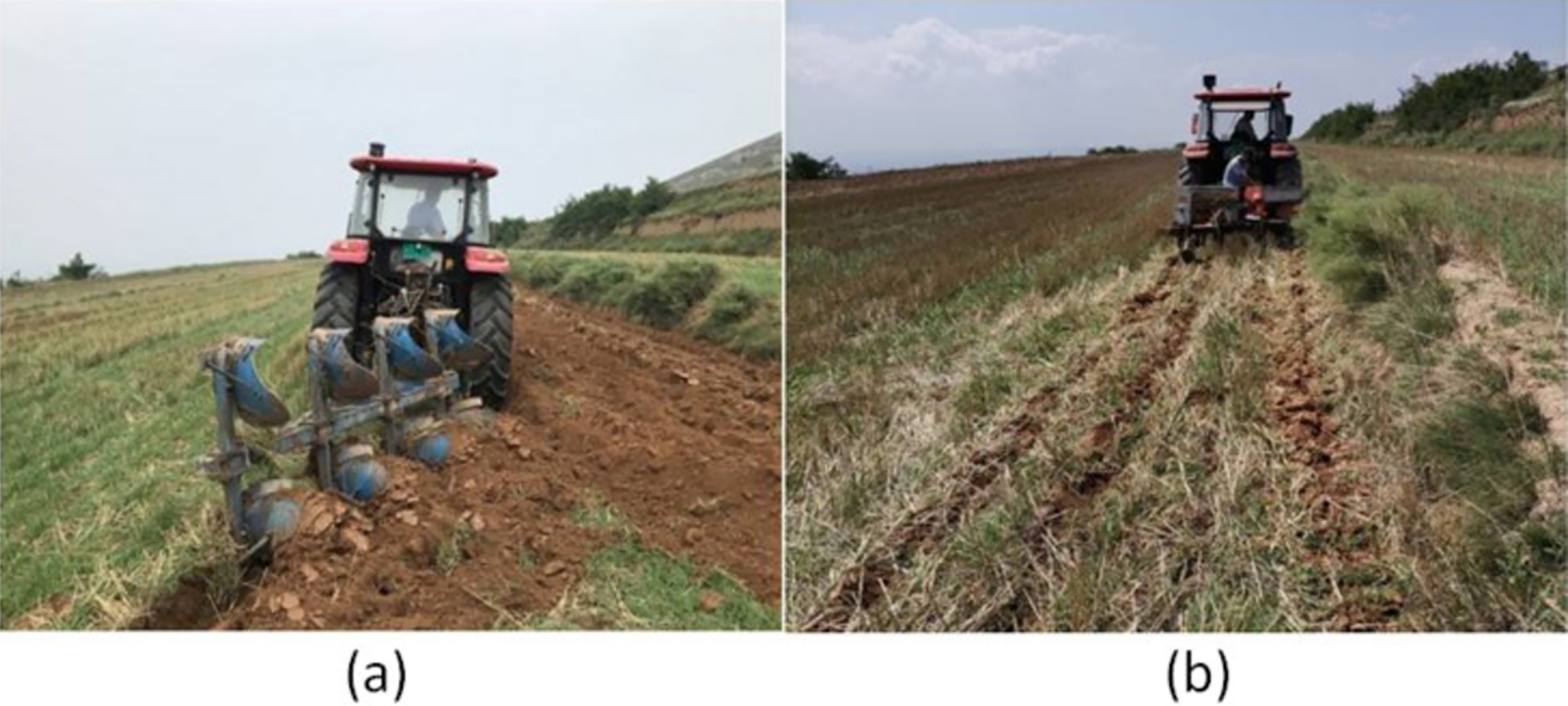
Field preparation at the experimental site of Shanxi Agricultural University. (A) deep ploughing, (B) subsoiling.

We tested winter wheat varieties was “Yunhan 20410”, which was provided by the Wenxi Agricultural and Rural Bureau. Within 10–15 days after harvesting wheat from the previous season, three tillage treatments were applied to the test field using agricultural equipment: (1) deep ploughing (DP): the depth was 0.25–0.30 m by applying deep tillage fertilization machine in fallow period, (2) subsoiling (SS): the depth was 0.30–0.40 m by subsoiling fertilization machine in fallow period, and (3) no tillage (NT): no tillage treatment was carried out during the fallow period as a control (Fig. 1). These were repeated three times on a 15 m × 6 m plot with a 0.3 m treatment interval. Winter wheat is harvested every year in June. Stubble height following harvest was approximately 0.2–0.3 m, which could effectively reduce water evaporation and increase soil organic carbon. Before sowing, nitrogen, phosphorus and potassium fertilizers were applied at the following concentrations: 150 kg ha^–1^ of nitrogen fertilizer (urea containing N 46%), 150 kg ha^–1^ of P_2_O_5_ (Calcium superphosphate containing P_2_O_5_ 16%) and 75 kg ha^–1^ of K_2_O (Potassium sulfate containing K_2_O 50%). The adoption of mechanical strip sowing included the application of 225 × 10^4^ plants·ha^–1^ of basic seedlings and 0.2 m of row spacing. During the season, weeding was practiced and no irrigation was applied.

### Sampling methods and measurements

#### Classification of precipitation types

In this study, we used the SPEI to determine the annual precipitation types. The SPEI is an index that is commonly used to describe the degree of drought (Stagge et al., 2016; Tirivarombo, Osupile & Eliasson, 2018; Faye, Grippa & Wood, 2019) by which the difference between precipitation and evapotranspiration in the fallow period (July–September) deviates from the average state. The calculations are as follows:

Step 1: Calculate the potential evapotranspiration (PET) according to the Thornth-Waite method adopted by Vicente-Serrano following:

(1) $$PE{T_i} = 16.0{\left( {\displaystyle{{10{T_i}} \over H}} \right)^A}$$

where: *PET*_*i*_ is the monthly potential evapotranspiration, *T*_*i*_ is the monthly average temperature, *H* is the annual heat index, and *A* is a constant.

Step 2: Calculate the difference between the monthly precipitation *P*_*i*_ and the evapotranspiration *PET*_*i*_:

(2) $${D_i} = {P_i} - PE{T_i}$$

Step 3: Normalize the *D*_*i*_ data sequence. The log-logistic probability distribution *F(x)* of the three parameters is used to normalize *D*_*i*_, and the SPEI value corresponding to each *D*_*i*_:

(3) $${\rm F}\left( x \right) = {\left[ {1 + \left( {\displaystyle{ \propto \over {x - \gamma }}} \right)\beta } \right]^{ - 1}}$$

where x is the value of the independent variable *D*_*i*_, *α* is the scale parameter, β is the shape parameter, and *γ* is the origin parameter.

Step 4: Standardize the cumulative probability density, that is: *p = 1 − F(x)*.

When the cumulative probability *p ≤ 0.5*, the SPEI value is calculated by:

(4) $${\rm SPEI} = {\rm w} - \displaystyle{{{c_0} + {c_1}w + {c_2}{w^2}} \over {1 + {d_1}w + {d_2}{w_2} + {d_3}{w_3}}}$$

(5) $${\rm w} = \sqrt { - 2ln\left( p \right)}$$

where *p* is the cumulative probability, the constant term *c*_*0*_ *= 2.515517*, *c*_*1*_ *= 0.802853, c*_*2*_ *= 0.010328, d*_*1*_ *= 1.432788, d*_*2*_ *= 0.189269, d*_*3*_ *= 0.001308*.

In Eq. (5), when *p > 0.5*, *p = 1 − p*, and the SPEI sign is negative.

Step 5: Divide drought levels according to McKee, Doesken & Kleist (1993) standards.

#### Soil water storage

Before plot preparation, a 3 m-deep profile pit was excavated, and soil samples were taken from a 0 to 3 m depth in 0.2 m increments using the cutting ring method described by Dam et al. (2005) before the sowing, wintering, jointing, anthesis, and maturity stages. We used a soil drill to take soil from each of the 0.2–3 m soil layers, with every 0.2 m considered a soil layer. The soil profiles were cut and levelled, and the samples were taken from the bottom to the top according to their designated level. A drying method was used to determine soil moisture in which soil samples were placed in a 105 °C oven after weighing and let stand for 72 h. The dry soil weight was then measured and the storage capacity of soil water storage was calculated:

(6) $$GSW = (wet\ soil\ mass - drying\ soil\ mass)/drying\ soil\ mass \times 100{\%}$$

(7) $$Soil\ water\ storage = GSW \times b \times SD \times {\it 10^3}$$

where *GSW* is the soil water content (%), *ρb* is soil bulk density of given soil layer (g cm^–3^), and *SD* refers to soil depth (m).

#### Grain yield and yield component

Fifty mature plants from each plot were randomly sampled from the inner rows to measure their yield components such as spike number, grains number per spike, and 1,000-grains weight. Grain yield was determined by harvesting all plants in the plot. After the plants were mechanically deshelled and the grains were air-dried, and the grain moisture meter (KETT PM-8188-A, Taiwan, Japan) was used to measure grain moisture content, and the actual yield was converted according to the national grain storage standard moisture content (12.5%).

### Statistical analysis

This study utilized meteorological data from the winter wheat growth stages between 2009 and 2018, and included the daily average, maximum and minimum temperatures, precipitation, accumulated temperature, and sunshine hours. When missing data exceeded a certain threshold, the data were eliminated. In other instances, we used the multi-year average data for the same date instead. Microsoft Excel 2010 and MATLAB R2014a were used for data processing and diagramming.

The soil water storage and winter wheat growth yield data was processed and statistically analyzed using SPSS 22 and SAS 8.6 (SAS Institute Inc., Cary, NC, USA). A two-way ANOVA was used to study the main effects and interactions of precipitation types and tillage methods on yield. When a significant interaction existed between precipitation year type, tillage, and yield, the least significant difference (LSD) method was used for variance analysis and independence t test, and the significance level was set to *α* = 0.05. Differences were considered statistically significant when *p* ≤ 0.05.

Factor analysis in SPSS 22 was used to select the eigen value with a characteristic root > 1. The meteorological factor corresponding to the highest load value after the maximum variance rotation was used as the uncontrollable modelling variable. We selected soil water storage, which has a significant correlation with yield and its other components, as the controllable modelling variable. The RF prediction model was constructed using MATLAB R2014a to complete data analysis and mapping.

### RF model construction and verification

**Figure 2:**
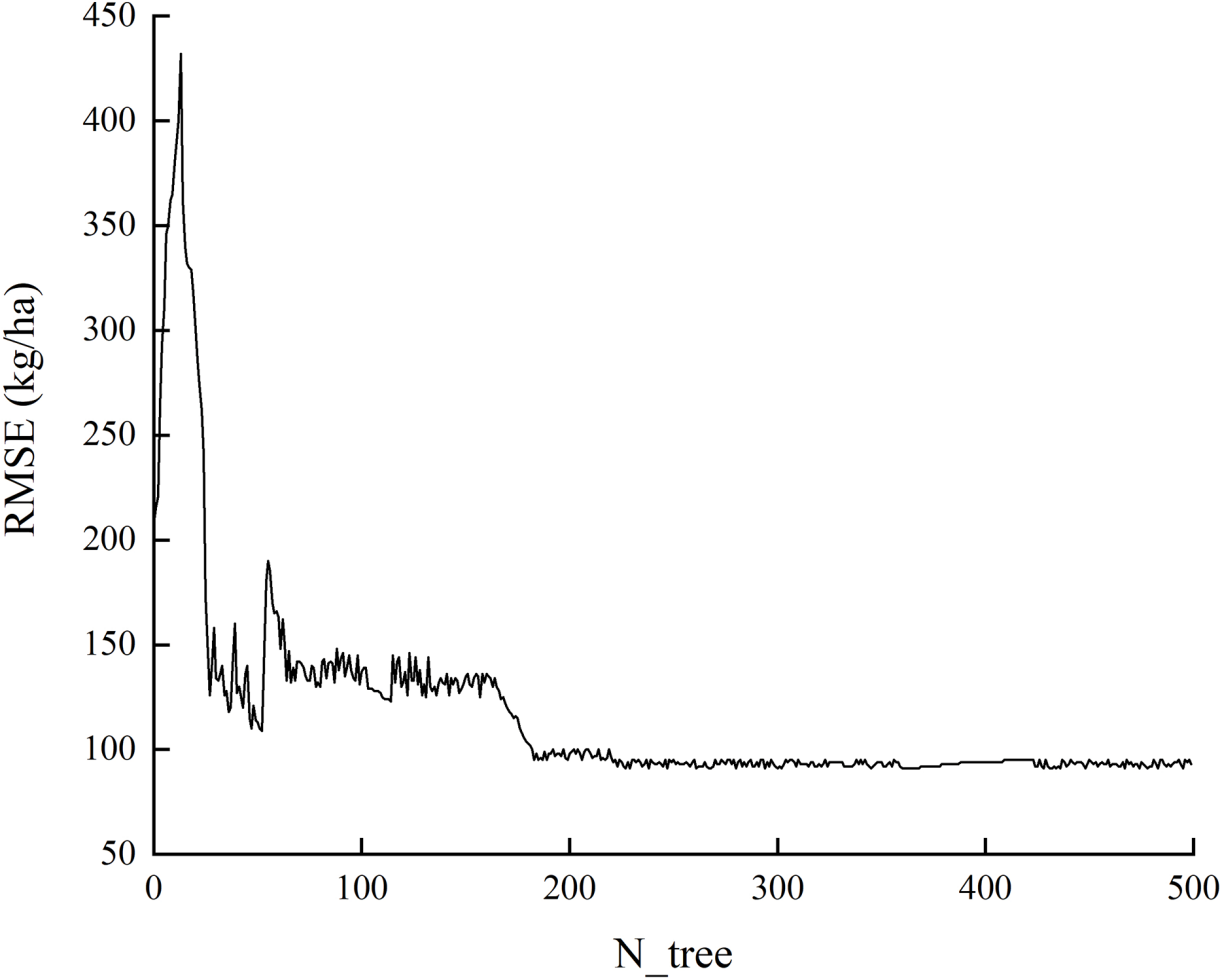
The relationship between the N tree and RMSE.

The RF algorithm is a widely-used statistical method first proposed by Breiman (2001). In this study, we used a repeated bootstrap sampling method to extract multiple samples from the initial samples by combining multiple relatively independent decision trees, establishing a “forest” of decision trees. For classification problems, the final classification result was determined through the voting of multiple tree classifiers. For regression problems, RF used double random sampling of samples and features to reduce the occurrence of over-fitting. The final prediction result was determined by calculating the average of the predicted values of multiple trees. In the RF, the number of decision trees (N) and the number of characteristic variables (m) needed to create branches were optimized according to the experimental results. First, we input the default parameter values N tree = 500 and m try = 5, and then used the root mean square error (RMSE) and out-of-bag (OOB) errors of the model to find the appropriate value that could best estimate the winter wheat yield. When N = 200, the RMSE roughly reached the minimum value and changed steadily (Fig. 2). To improve our calculation efficiency, we set the N tree to 200 and m try to 5.

Typically, crop yields are divided into three parts: trend yield, climate yield, and an error component. Trend production refers to the long-term stable increase in production due to technological progresses at the productivity level. Climate production refers to the short-term, fluctuating production caused by a change in meteorological factors that may increase or decrease. The error component refers to the yield affected by accidental factors, such as disease, pests, and social unrest, among other contributors. Because the proportion of the latter is very small in actual production, it is typically not considered (Wang et al., 2018). The formula is:

(8) $$y = {y_t} + {y_c} + e$$

where *y* is the measured yield (kg ha^–1^), *y*_*t*_ is the trend yield (kg ha^–1^), *y*_*c*_ is the meteorological yield (kg ha^–1^), and *e* is the error component.

The meteorological yield was obtained by subtracting the trend yield from the actual yield in each year, and the relative meteorological yield was obtained by dividing the meteorological yield by the trend yield. The relative meteorological yield reflects the yield fluctuation caused by the meteorological difference between years. Three yield prediction models were constructed using unit yield, meteorological yield, and relative meteorological yield as the target variables in the RF. The formula is:

(9) $${y_w} = \displaystyle{{y - {y_t}} \over {{y_t}}}$$

where *y*_*w*_ is the relative meteorological yield and *y*_*t*_ is the trend yield (kg ha^–1^).

To estimate the accuracy of RF predictions, we used the coefficient of determination (R^2^), RMSE and mean relative error (MRE) between the measured and predicted values to assess the model performance (Childs, Coffey & Travis, 2007; Pouladi et al., 2019). The formulas are:

(10) $${R^2} = 1 - \displaystyle{{\mathop \sum \nolimits_{i = 1}^n {{\left( {{S_i} - {O_i}} \right)}^2}} \over {\mathop \sum \nolimits_{i = 1}^n {{\left( {{O_i} - {O_m}} \right)}^2}}}$$

(11) $$RMSE = \sqrt {\displaystyle{{\mathop \sum \nolimits_{i - 1}^n {{\left( {{S_i} - {O_i}} \right)}^2}} \over n}}$$

(12) $$MRE = \displaystyle{1 \over n}\mathop \sum \limits_{i = 1}^n \displaystyle{{\left| {{S_i} - {O_i}} \right|} \over {{S_i}}}$$

where *S*_*i*_ is the model’s predicted value, *O*_*i*_ is the measured value, *O*_*m*_ is mean of the measured value, *i* is the number of samples, and *n* is the total number of samples.

## Results

### Effect of cultivation on yield for different fallow period precipitation types

#### Precipitation type and winter wheat yield

We used the SPEI as the classification standard of precipitation year types. Fallow period precipitation is critical for water use efficiency and high-yield/upland farming systems for dryland crops. During the study period, the average precipitation in the test site during the fallow period was 283.53 mm, accounting for 59.45% of the annual precipitation. The fallow period of winter wheat at the test site was from July to September. We calculated the SPEI values across a 3-month scale and selected the SPEI values corresponding to September of each test year (this was calculated based on the cumulative precipitation and potential evapotranspiration from July to September). We then determined the precipitation year type according to the SPEI value on the 3-month scale and categorized the 2009–2018 test points into normal and drought types. There were 5 normal years and 5 drought years (Table 3).

**Table 3:** Annual precipitation type classification based on the SPEI. The 2009–2018 test points were categorized according to their SPEI values across a 3-month scale into normal and drought precipitation types.

Precipitation types	SPEI-3	Year
Normal	−0.5 < SPEI	2009, 2010, 2011, 2012, 2014
Drought	SPEI ≤ −0.5	2013, 2015, 2016, 2017, 2018

Our analyses of variance between precipitation types, tillage, yield, and yield components all passed the 0.05 significance test. The effects of different tillage treatments on yield and other components under different precipitation types are presented in Table 4. Fallow tillage achieved higher yields and yield components than NT under different precipitation types. Under the normal type, DP and SS produced significantly different yields, spike numbers, and grains number per spike, and the yield of winter wheat under DP was significantly higher than SS. SS had the highest yield (5,149.10 kg ha^–1^), grains number per spike (32.44 grains per spike), and 1,000-grains weight (40.75 g) under drought precipitation conditions. DP’s yield and its components under drought years were significantly higher than those under normal years, and the yield obtained by SS showed no significant difference across the different precipitation types.

**Table 4:** Tillage method and winter wheat yield, and yield components under different precipitation types.

Precipitation types (P)	Tillage (T)	Yield (kg ha^−1^)	Spike number (10^4^ ha^−1^)	Grains number per spike	1,000-grains weight (g)
Normal	DP	4,529.12^b^	482.21^a^	26.32^d^	40.30^b^
	SS	4,385.94^c^	464.02^b^	25.55^e^	40.07^c^
	NT	3,428.15^e^	402.30^d^	23.75^f^	37.54^f^
Drought	DP	5,149.10^a^	478.22^a^	32.44^a^	40.75^a^
	SS	4,437.41^c^	447.08^c^	31.70^b^	39.27^d^
	NT	4,070.68^d^	397.33^d^	30.05^c^	38.45^e^

ANOVA table (LSD protected, *p* ≤ 0.05)				
F_T_	90.92*	19.52*	50.94*	3.28
F_P_	246.65*	42.67*	666.78*	0.23
F_T_ × F_P_	−95,001.58*	−1,757,504.64*	−10,946.57*	−509,247.08*

**Notes:**

The differences of winter wheat yield, spike number, grain number per spike, and 1,000-grains weight across the two precipitation types (normal type and drought type). Letters a–f indicate the significant differences between the treatments (*p* ≤ 0.05) determined by LSD test. The same letter indicates that there was no significant difference between treatments.

* The F test was significant *p* ≤ 0.05.

#### Tillage and winter wheat yield during the fallow period

**Figure 3:**
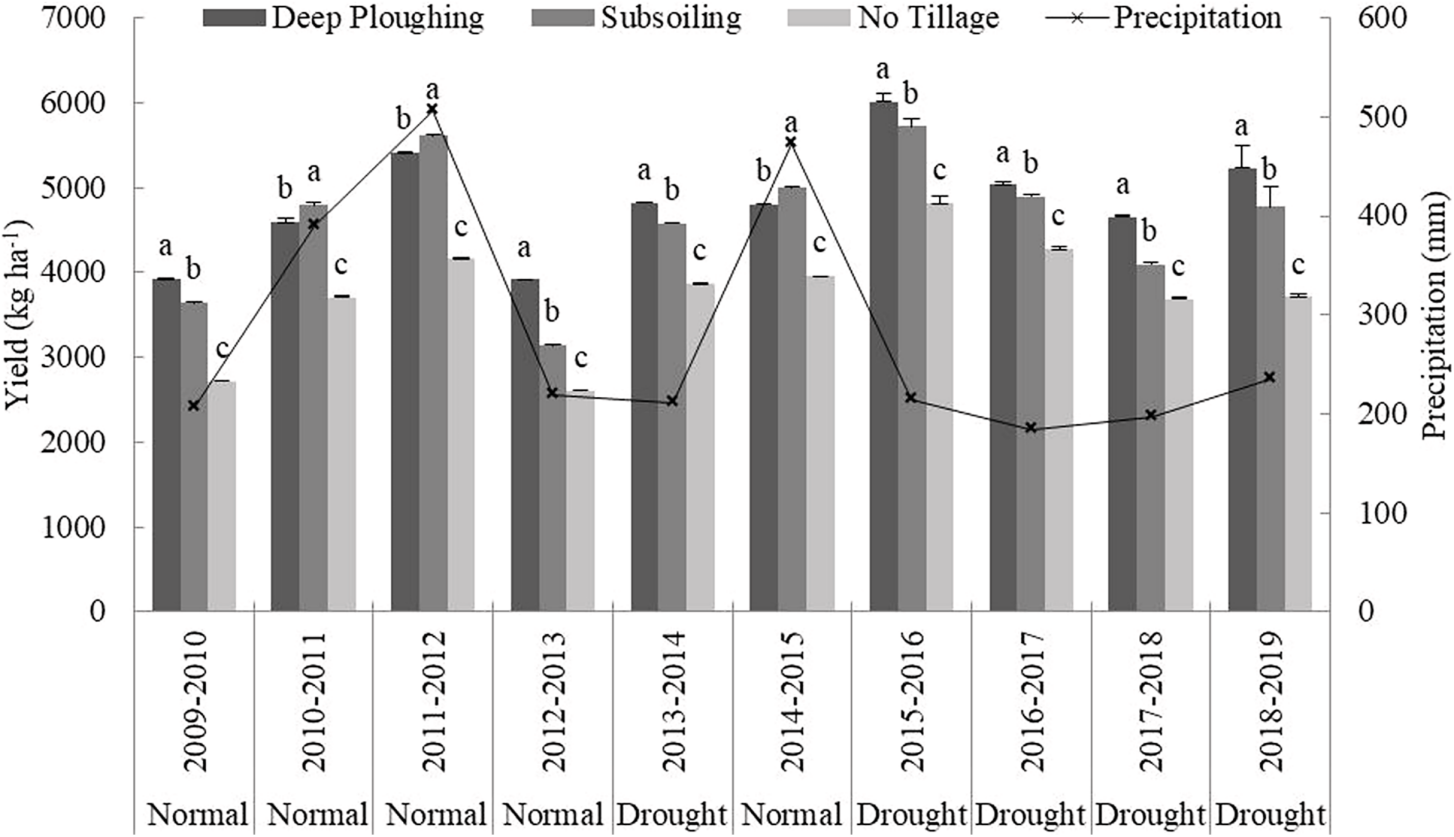
Impact of different tillage methods on yield. a, b, and c represent the significant differences obtained by the LSD method across different farming methods. The line represents the precipitation in the fallow period from 2009 to 2018.

When compared with NT, tillage during the fallow period significantly increased the test field yield (Fig. 3). DP significantly increased wheat yields under different precipitation types, especially drought years, and the highest yield was obtained under DP in 2015 (6,009 kg ha^–1^). This was significantly higher than the yield of fields treated with SS. The yield of SS in 2010, 2011, and 2014 was higher than that of DP, which may be due to the precipitation during those fallow periods being slightly higher than that of other normal years, causing an increase in yield. This indicates that precipitation and tillage during the fallow period are closely related.

### Winter wheat yield prediction based on the RF algorithm

#### Selection of meteorological factors

In this study, we selected 14 meteorological indices, including precipitation in the fallow period, average temperature, daily maximum temperature, daily minimum temperature, accumulated temperature and sunshine duration, precipitation, average temperature, and accumulated temperature and sunshine duration during the two growth stages (sowing-jointing and jointing-anthesis). After analyzing the meteorological factors, a total of four common factors (Table 5) were extracted using a principle eigen value > 1. The cumulative explanatory power of variance was 85.51%, and factor 1 and factor 2 had the largest explanatory power (65.11%). After the maximum variance rotation of the meteorological factors, we selected the meteorological indices with the largest load values among the four factors (Table 6). The selected meteorological factors included fallow period precipitation, sowing-jointing stage precipitation, daily minimum temperature, average temperature, and average temperature during the sowing-jointing stage, sunshine hours during the jointing-anthesis stage, and six other items.

**Table 5:** Cumulative contribution rate and eigen values. Using factor analysis and the principle of eigen value, four common factors were extracted.

Components	Initial eigen value		
	Eigen value	Variance %	Cumulative variance %
1	5.01	36.43	36.43
2	4.02	28.68	65.11
3	1.70	12.14	77.25
4	1.16	8.26	85.51

**Table 6:** Rotation factor’s load amount. Fourteen meteorological factors were selected according to their rotation factor load, and the high load values were selected as meteorological factors to participate in modeling.

Factor	Fallow period precipitation	Stage 1 precipitation	Stage 2 precipitation	Accumulated temperature during growth period	Average temperature during the growing period
1	**0.89**	−0.13	−0.89	−0.78	−0.07
2	0.15	0.15	−0.28	0.54	0.97
3	0.32	−0.03	0.03	0.03	−0.08
4	0.29	**0.96**	0.03	0.27	0.10
Factor	Daily maximum temperature	Daily minimum temperature	Stage 1 average temperature	Stage 2 average temperature	Sunshine during growth period
1	0.04	−0.18	−0.49	0.27	0.80
2	−0.60	0.19	0.71	**0.97**	0.07
3	0.68	**0.88**	−0.06	0.03	−0.22
4	−0.07	0.03	−0.03	0.21	−0.28
Factor	Stage 1 sunshine	Stage 2 sunshine	Stage 1 accumulated temperature	Stage 2 accumulated temperature	
1	0.61	**0.89**	−0.86	0.17	
2	0.51	−0.02	0.48	0.77	
3	−0.06	−0.33	−0.01	0.39	
4	0.23	−0.43	−0.03	−0.03	

**Note:**

Stage 1 is Sowing-Jointing stage; stage 2 is Jointing-Anthesis stage. Bold letters indicate high loads.

#### Correlation between soil water storage and yield

After analyzing the correlation between the water storage of each growth stage, yield, and its components, the relationship between soil water storage in each growth stage and its yield (including its components) under different precipitation types was clear (Table 7). The 0–3 m soil water storage of the jointing and anthesis stage was significantly correlated to the yield, spike number, and grains number per spike. Although the correlation between soil water storage and grains number per spike during the sowing stage was not significant, there was a certain correlation between soil water storage and grains number per spike during the other growth stages. Soil water storage during the sowing stage correlated with yield, and significantly correlated with spike number. Across different growth stages, the correlation between the 1,000-grains weight and soil water storage was not significant.

**Table 7:** Pearson simple linear correlation between soil water storage, yield, and yield composition. The correlation between soil water content, yield, and yield components across different growth stages.

Soil water storage	Yield	Spike number	Grains number per spike	1,000-grains weight
Sowing stage	0.78*	0.93**	0.46	0.52
Jointing stage	0.95**	0.90**	0.81**	0.62
Anthesis stage	0.92**	0.89**	0.88**	0.47
Maturity stage	0.75*	0.84**	0.77*	0.33

**Notes:**

* *p* < 0.05.

** *p* < 0.01, The same below.

During the modelling process, we considered the soil water storage during the sowing and jointing-anthesis stages important variables affecting yield, spike number, and grains number per spike. In this study, soil water storage during the growth period was considered an important variable in modelling, and by combining modelling with uncontrollable meteorological factors, we could predict the yield and its components across different tillage methods.

#### Comparing prediction results of different target yields

**Figure 4:**
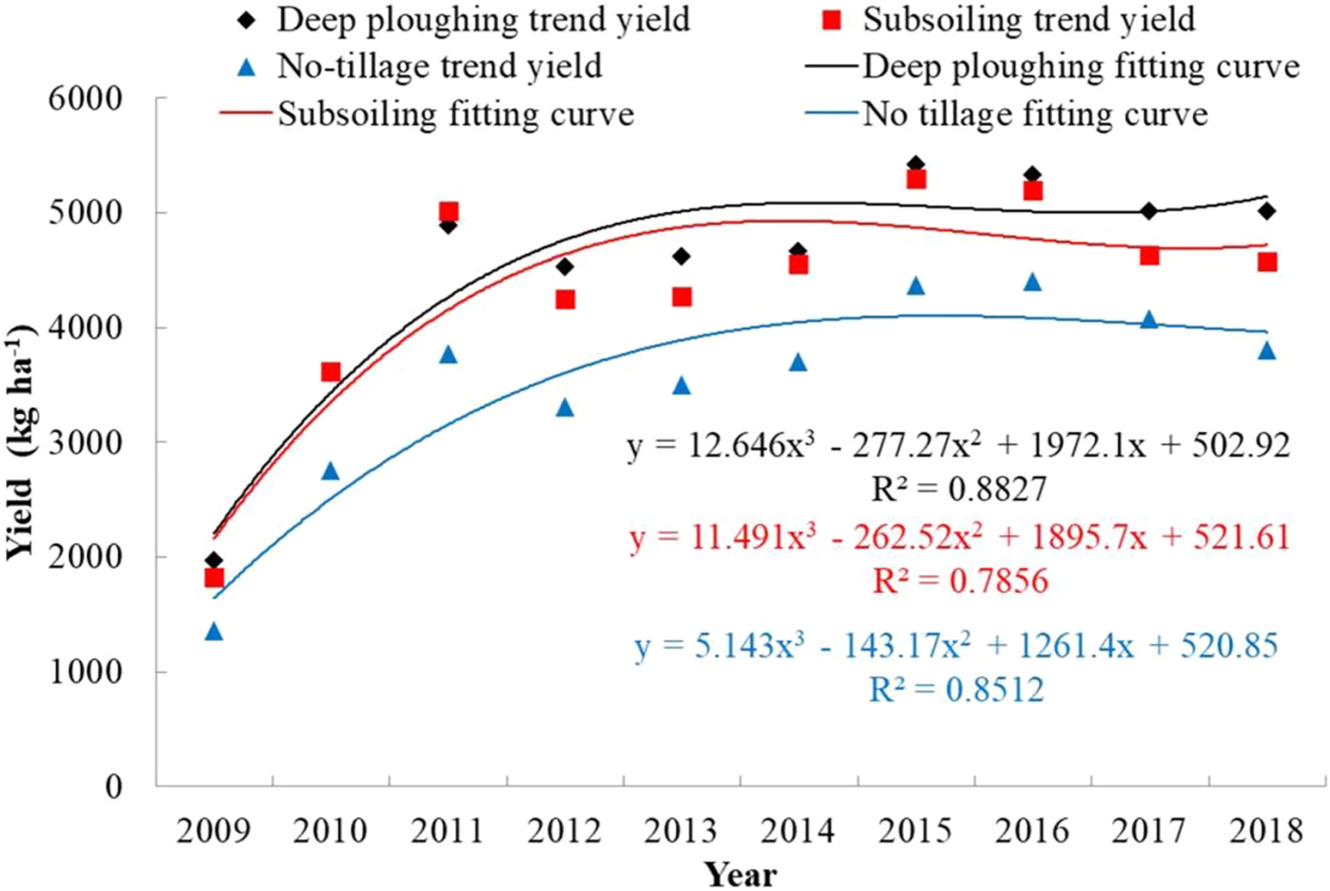
Trend yield and fitting of winter wheat under different tillage treatments. The black diamond represents the trend yield of DP, the red rectangle represents the trend yield of SS, and the blue triangle represents the trend yield of NT. The black curve and black formula represent DP’s trend yield fitting curve and its fitting formula; the red curve and red formula represent SS’s trend yield fitting curve and its fitting formula for subsoiling; and the blue curve and blue formula represent NT’s trend yield fitting curve and its fitting formula.

In the scenario where the trend yield has not been eliminated, the maximum value of the training samples in the yield per unit area model was higher than that of the latter test set years, which resulted in higher prediction results. Theoretically, the meteorological and relative meteorological yield are only affected by meteorological conditions, but their values cannot be obtained directly by measurement--they depend on the selection of a detrend method. In our previous study, we compared a variety of methods for separating trend production. When building the model, we used 552 sets of data from 2009 to 2016 as training samples and, based on experience and parameter optimization, we set the RF algorithm parameter N tree to 200 and m to 5. We used 138 sets of data from 2017 to 2019 as validation samples to validate the model. Ultimately, the 3-year linear and sliding average method was selected to separate trend production. The three farming treatments were fitted with polynomials, the fitting effect was better, and the fitted curves of DP, SS, and NT had R^2^ values of 0.88, 0.79, and 0.85, respectively (Fig. 4). The yield was detrended based on the 3a moving average method to obtain the meteorological and relative meteorological yield. To some extent, it eliminated the interannual impact of yields and highlighted the impact of meteorological factors on yield.

**Figure 5:**
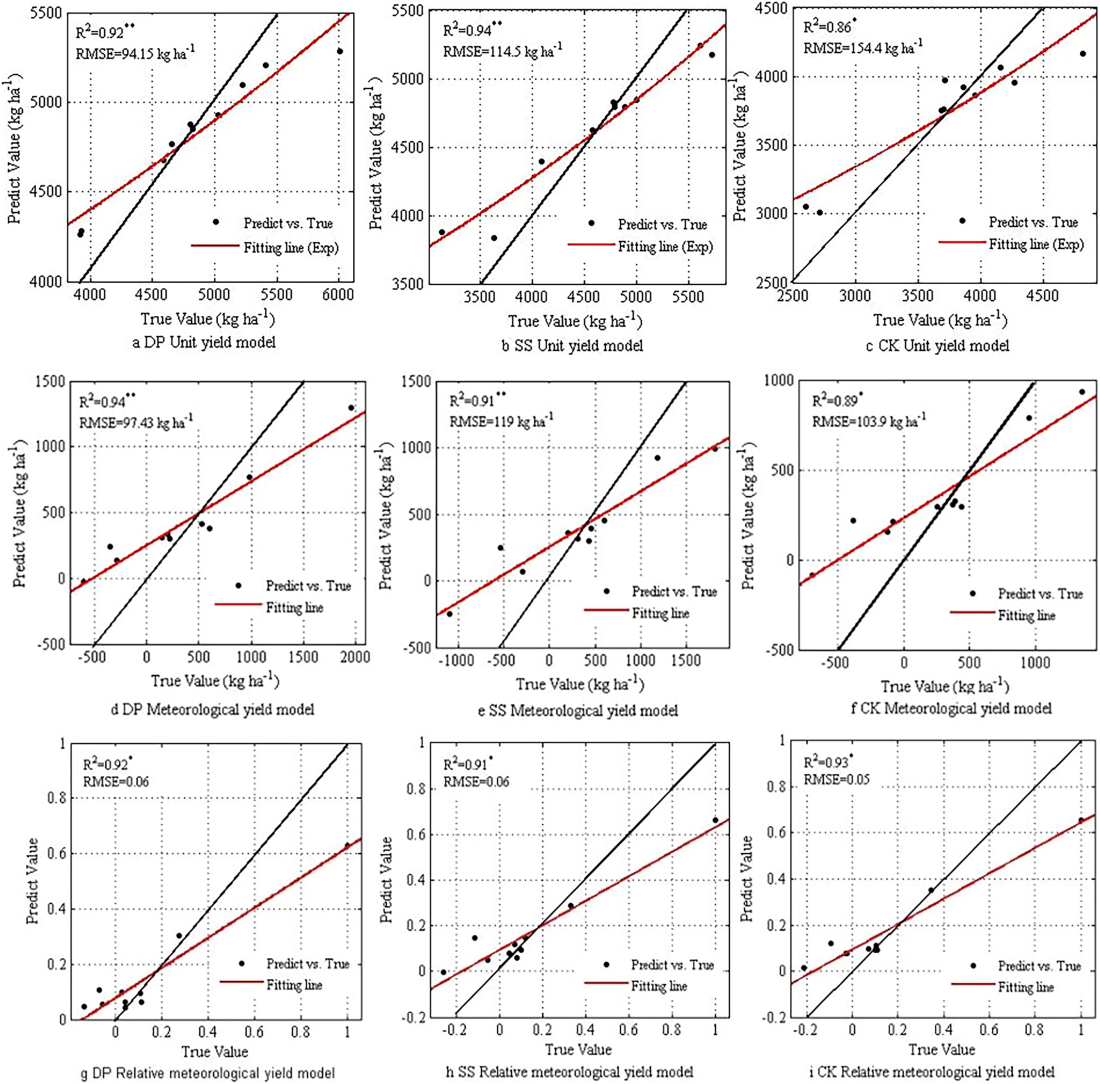
Comparing RF prediction results of different target variables.

More than 56.67% of the sample forecast results were higher than the true value, and there were many phenomena where the yield forecast value under DP and NT was higher than the true value (Fig. 5). Following the tillage during the fallow period, the DP and SS yield prediction models’ R^2^ both passed the significance test, and the RMSE reached 94.15 kg ha^–1^ and 114.5 kg ha^–1^, respectively. The samples were mostly concentrated at 4,500–5,000 kg ha^–1^. The NT R^2^ between the predicted yield value and the measured value was 0.86. According to the significance test threshold of *p* ≤ 0.05, the RMSE was 154.4 kg ha^–1^, which was slightly higher than the yield prediction result following the fallow period. NT’s yield prediction was not as high as the yield prediction result under the tillage treatment during the fallow period.

The meteorological production forecast was similar to the unit yield forecast. DP and SS’s meteorological production forecast R^2^ was above 0.9, and the RMSE were 97.43 kg ha^–1^and 119 kg ha^–1^, respectively. The intersection of the SS fitting line and the 1:1 line was closer to the scale 0. In terms of relative meteorological yield, there was a small error of determination coefficient between tillage and NT during the fallow period, indicating that the RF was highly fitted in terms of relative meteorological yield. It also showed its ability to influence the inter annual production technology level of the yield. The fitting line was closest to the 1:1 line, which shows that under the influence of different meteorological conditions, the relative meteorological yield had a better fitting effect than the unit yield model.

#### Comparing forecast results of yield and yield components

We used the RF model to predict the yield under different tillage methods, and the error between the predicted value and the true value was small, ranging from 0 to 23.46% (Table 8). Under different precipitation types, the average relative error was 5.66%, which was slightly higher than the true value. The average relative error of tillage treatment during the fallow period was less than that of NT. This shows that the RF algorithm can better predict yield under tillage in the fallow period. During the test period, the average yield of DP was higher than the predicted yield by 15.83 kg ha^–1^, with an average relative error of 4.51%. The average yield of SS was lower than the predicted yield by 16.29 kg ha^–1^ with an error of 5.98%. The error between the predicted value and the true value of the average yield under NT was 6.49%. Generally, the error value was small and the prediction result was better in field production. The predicted value of 60% of the yield samples in the normal years was higher than the true value. The DP, SS, and NT values were 133.14 kg ha^–1^, 80.8 kg ha^–1^, and 122.19 kg ha^–1^ higher, respectively. About 53.33% of the predicted yields in drought years were higher than the measured values, and the average predicted performance of tillage during the fallow period was better than that of NT.

**Table 8:** RF prediction results and errors under different tillage methods.

Type	Year	Deep ploughing			Subsoiling			No-tillage		
		True value (kg ha^−1^)	Predict value (kg ha^−1^)	Error (%)	True value (kg ha^−1^)	Predict value (kg ha^−1^)	Error (%)	True value (kg ha^−1^)	Predict value (kg ha^−1^)	Error (%)
Normal	2009	3,923.57	4,283.53	9.17	3,639.82	3,835.16	5.37	2,714.96	3,012.48	10.96
	2010	4,588.15	4,676.75	1.93	4,794.56	4,791.82	0.06	3,705.67	3,761.26	1.50
	2011	5,412.04	5,208.05	3.77	5,612.45	5,241.97	6.60	4,155.60	4,065.82	2.16
	2012	3,915.32	4,263.74	8.90	3,140.25	3,877.02	23.46	2,608.30	3,051.83	17.00
	2014	4,806.55	4,879.21	1.51	4,999.96	4,845.06	3.10	3,956.22	3,860.30	2.42
	MEAN	4,529.12	4,662.27	2.94	4,437.41	4,518.21	1.82	3,428.15	3,550.34	3.56
Drought	2013	4,818.74	4,850.41	0.66	4,575.40	4,626.02	1.11	3,866.73	3,919.66	1.37
	2015	6,009.75	5,483.64	8.75	5,719.08	5,175.11	9.51	4,812.00	4,170.89	13.32
	2016	5,032.00	4,925.96	2.11	4,892.00	4,789.95	2.09	4,274.00	3,958.87	7.37
	2017	4,657.53	4,767.79	2.37	4,093.03	4,398.23	7.46	3,689.52	3,753.52	1.73
	2018	5,227.48	5,093.71	2.56	4,775.68	4,824.75	1.03	3,711.17	3,974.16	7.09
	MEAN	5,149.10	4,984.30	3.20	4,811.04	4,762.81	1.00	4,070.68	3,955.42	2.83

**Figure 6:**
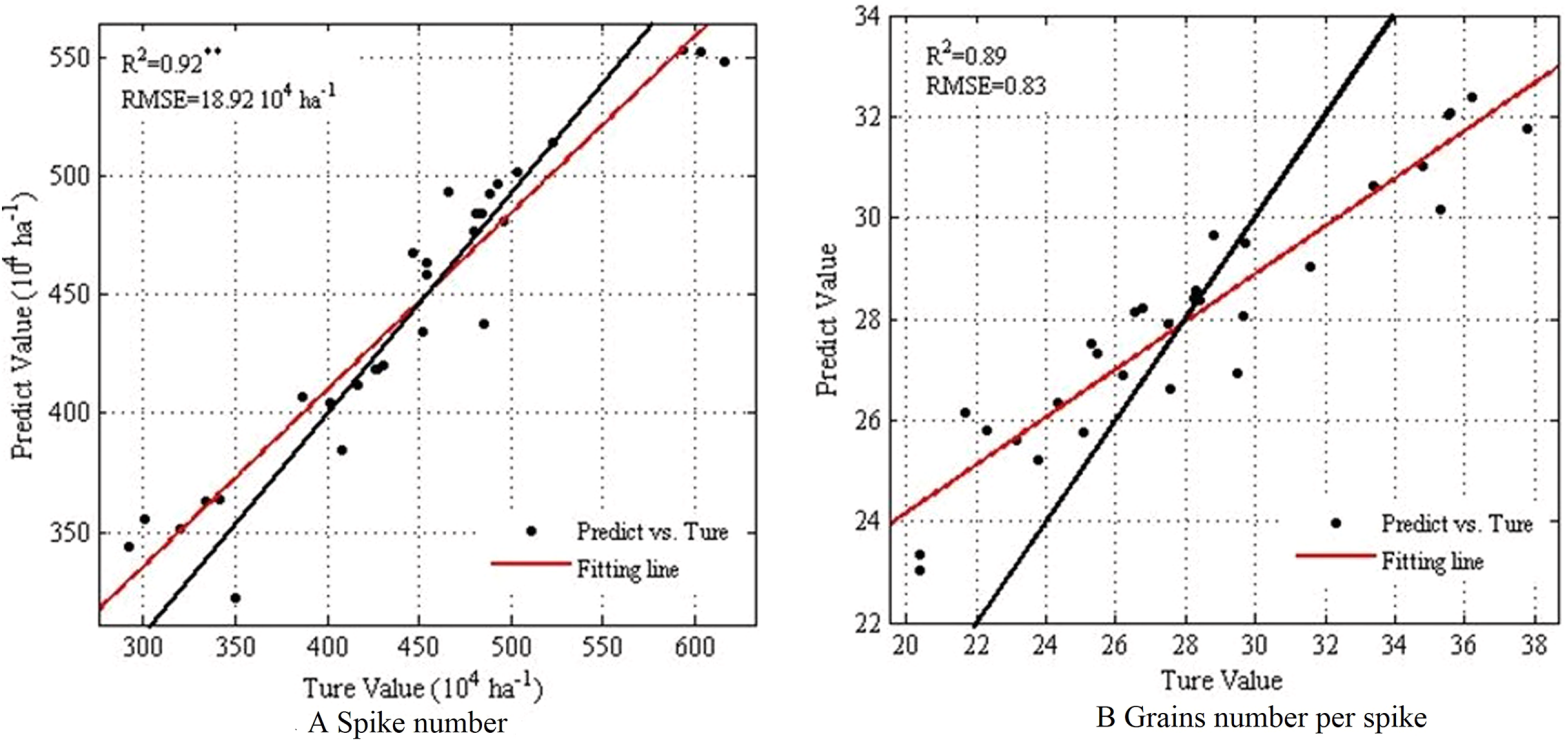
Comparing RF prediction results of spike number and grains number per spike.

Additionally, RF was used to predict the number of spikes and grains number per spike under different tillage methods. The spike number prediction model R^2^ passed the significance test: R^2^ was 0.92 and the RMSE was 18.92 10^4^·ha^–1^ (Fig. 6). The grain number samples were in the range of 400–500 10^4^·ha^–1^, which was close to the 1:1 line. Additionally, the average prediction error was 4.8%, indicating that the prediction results had a high degree of credibility. The sample distribution of grain number per spike was more scattered, with an R^2^ and RMSE of 0.89 and 0.83, respectively, and the predicted value of most samples concentrated in the range of 22–30. The average prediction error was 7.53%.

**Figure 7:**
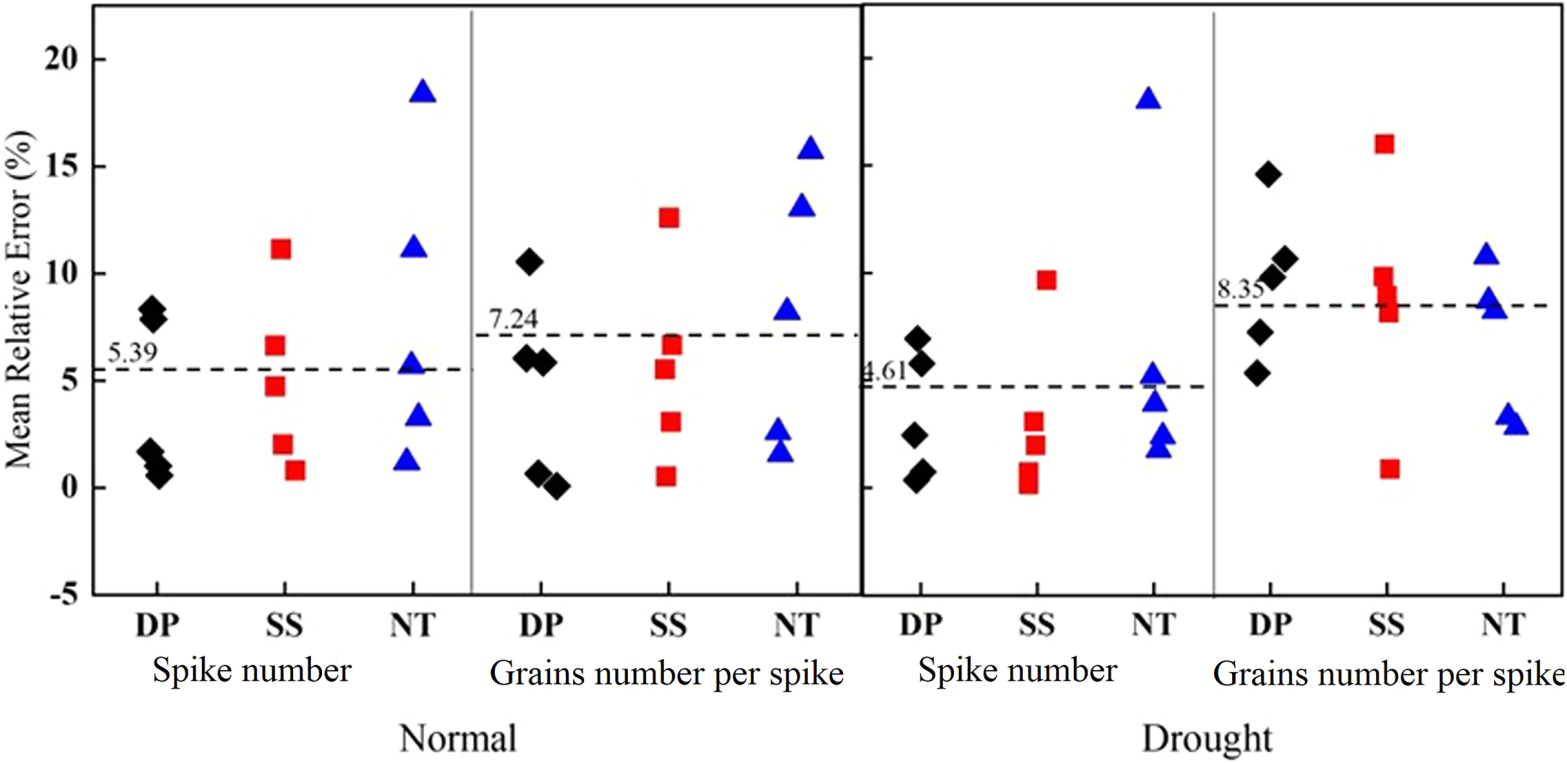
Comparison of spike number and grains number per spike errors under different tillage method. The dotted line represents the average value.

By comparing the prediction errors of spike number and grains number per spike under different tillage methods, we found that the prediction accuracy of tillage during the fallow period was higher than that of NT (Fig. 7). Under different precipitation types, the average prediction error of spike number was 4.8%. This prediction result was better than that of grains number per spike (with an average prediction error of 7.53%), and the grains number per spike had better prediction results under normal year types. The predicted farming performance across different years’ fallow periods was better than that of NT, and the true value was closer to the predicted value. The spike number prediction in drought years was better than that of normal years, and the grains number per spike was better in normal years. The results show that the prediction method obtained lower error when predicting spike number and grains number per spike of winter wheat under tillage during the fallow period, and the prediction results were closer to the true value.

After fitting the measured value and predicted value, RF showed good prediction ability for winter wheat yield, spike number, and grains number per spike after fallow period tillage under different precipitation types in the studied dryland region. We considered it suitable for the prediction of winter wheat yield and its component factors in the dryland wheat region. The yield prediction results were the most accurate, followed by spike number and grains number per spike, whereas the 1,000-grains weight was not suitable for the model.

## Discussion

### Feasibility of the precipitation year type classification based on the SPEI

Dryland winter wheat mainly relies on natural precipitation, but in the Loess Plateau, precipitation mainly occurs during the fallow period. This leads to lower levels of precipitation during the growth period, and a decline in wheat production (He et al., 2016). In conjunction with climate change, the variability of precipitation and the number of high temperature days increase (IPCC, 2014; Zhang et al., 2016). Fallow precipitation in different cultivating years is quite variable, which leads to variable availability in soil moisture during the growth period of winter wheat across different years. It is necessary to classify different cultivating years in order to help farmers choose appropriate field management measures (Wang et al., 2020). Regarding the classification of the year type, agricultural scientists often use the annual average precipitation as the basis for the classification of the year type. There are many meteorological factors that affect crop growth. If only water is considered, the contribution of water factors to yield will be overestimated to a certain extent. According to the precipitation and temperature during the fallow period, the year type classification was more accurate and the data calculation was more balanced, especially considering the climatic characteristics of China’s Loess Plateau. We used the SPEI and McKee, Doesken & Kleist (1993) drought/humidity classification standard to classify the wheat planting years into two types: normal and drought (Table 3). Previously, Sun et al. (2018) classified 2009, 2012, and 2013 as drought-type years according to the average annual precipitation and precipitation during the fallow period. Statistics dates shows that the fallow precipitation in 2009 and 2012 were 206.2 mm and 218.9 mm, respectively. In addition to precipitation, plus the effects of temperature and evapotranspiration, SPEI during the fallow period is calculated to be >−0.5, which is regarded as normal year in this study; 2010, 2011, and 2014 were considered non-drought years, which was consistent with the work of Ren et al. (2019). The classification method is more comprehensive, can avoid overestimating the influence of water on crop yield, and is suitable for year type classification in loess Plateau region.

### Fallow tillage can affect soil water storage and yield formation

Reasonable tillage measures can significantly improve the soil environment and facilitate the sustainable development of cultivated land resources (Huang et al., 2020). Past studies have used tillage measures that accumulate precipitation to increase the water needed for crop growth in the rain-fed farming area of Loess Plateau (Ren et al., 2019; Yu et al., 2021), including conservation tillage (either reduced tillage or no tillage), SS, and DP (Hou et al., 2012). Conservation tillage reduces soil loss by providing protective mulch. This practice promotes water balance by reducing evaporation from the soil surface and improves soil water-holding capacity by improving soil structure (Peng et al., 2020). Reduced tillage maximizes water conservation compared to subsoiling (Yin et al., 2021). Basir et al. (2017) conducted tillage experiments in the wheat continuous cropping area in Pakistan and found that under the soil conditions, shallow tillage (0–0.1 m), residual mulching of maize and application of nitrogen fertilizer could improve the productivity of wheat crops. Our experiment does not involve other cultivation methods at present, only considering the effect of tillage methods on yield. Previous studies have also shown that the positive effect of fallow tillage on soil moisture is not limited by the type of crop. In addition to wheat, it also has a positive effect on increasing the soil moisture required for the growth of cereal crops (Hou & Li, 2018; Santín‐Montanyá et al., 2020). Tillage during the fallow period can significantly increase the soil water storage during the sowing-joining and jointing- anthesis stages (Xue et al., 2019). SS can significantly increase soil water storage at a depth of 0–3 m. This conclusion was coincided with the results of Liang et al. (2019) from other areas of the Loess Plateau. Soil water storage is directly affected by natural precipitation (Feng et al., 2017; Yu et al., 2020). Our research shows that the soil water storage across different growth periods will affect the yield and components of wheat at harvest (Table 8), which was also confirmed by Xue et al. (2019). Our team’s previous research has reported on the effect of soil water storage in different soil layers at each growth period on the number of ears, grains per ear, and plant nitrogen accumulation (Sun et al., 2018; Xue et al., 2019; Ren et al., 2019). Therefore, when constructing a reasonable yield prediction model, we give priority to the soil water storage capacity of 0–3 m in each growth period as a parameter. The prediction results show that the method is feasible and the prediction error is small. We hope that in the future, our research will be specific to the water storage capacity of different soil layers, In order to improve the output forecast results and improve the accuracy of the forecast.

### RF’s performance in predicting winter wheat yield after tillage during the fallow period

The growing recognition of the role that food security plays in stabilizing and developing economies also highlights the importance of forecasting crop yields (Liu & Basso, 2020). Manual production estimation is tedious and can produce significant errors. In exploring more scientific and precise methods, scientists have begun to use crop growth models and machine algorithms to study the crop growth process and yield (Liaqat et al., 2017; Mehta et al., 2018; Ahmadian et al., 2019; Li et al., 2021). At present, many crop growth models have been applied, such as APSIM, DSSAT, WOFOST, etc., which can accurately simulate crop growth stage by changing the parameter Settings of crop growth stage (Rosa, Souza & Tsukahara, 2020; Zhao et al., 2020). In future studies, we can compare the accuracy of different crop growth models in yield simulation and modify the models to be suitable for local crops. The Web of Science database includes 24 research manuscripts that use machine learning algorithms, crop yield prediction, and other related keywords from 2017 to 2021. Among these, 14 research manuscripts (File S3) showed that the most commonly-used machine algorithms were the backpropagation neural network (BPNN), decision tree (DT), Gaussian process regression (GPR), k-nearest neighbor regression (KNN), linear discriminant analysis (LDA), quadratic discriminant analysis (QDA), support vector machine (SVM), decision trees, and RF (Sharifi, 2020; Pant et al., 2021; Kanwal et al., 2021). A total of 75% of these studies identified that the RF model performed better in prediction, combining weather parameters, remote sensing data, and field observation data when completing the construction of the model to predict yield. Li et al. (2021) used RF to predict the yield of three major grain crops in China based on climate, vegetation indices, and soil properties, and found that the RF model performed well in predicting the grain yield. This was consistent with our research findings and confirmed the performance of RF in crop yield prediction on the Loess Plateau. Under the background of climate change, the influence of meteorological factors on crop growth is increasing. In order to maintain high yield, it is necessary to optimize field management measures. In addition to the fixed-point experiment, multi-point comparative experiment can be carried out in other areas of the Loess Plateau, and different water and fertilizer experiments can be designed from the spatial regional scale to predict the yield with the help of crop growth model, which can provide reference for the prediction of winter wheat yield in the Loess Plateau.

## Conclusions

In summary, our results from 10 years of field experiments showed that when compared to NT, tillage during the fallow period significantly increased the crop yield. The classification of different planting year types according to the SPEI of the fallow period was more in line with the climate background of dryland wheat. DP during the fallow period achieved a higher yield. RF was suitable for predicting the yield, spike number, and grains number per spike of wheat in dryland under tillage during the fallow period with small errors and good performance. Out of all the research indices included in this study, DP was the ideal tillage method during the fallow period. It is essential to construct an early-stage RF yield prediction model in order to formulate appropriate field management measures and to provide a basis for the implementation of grain production systems, which will benefit agricultural managers and farmers in this region.

## Supplemental Information

10.7717/peerj.12602/supp-1 Supplemental Information 1 Provide relevant original data in the manuscript. Supplement 1 includes meteorological data such as precipitation, air temperature, accumulated temperature and sunshine duration from 2009 to 2019, yield data after tillage in different fallow periods and data needed for Random Forest modeling

10.7717/peerj.12602/supp-2 Supplemental Information 2 Random Forest modeling code and modifiable diagrams in the manuscript are provided. The result graph and code predicted by the Random Forest algorithm appear in Supplement 2

10.7717/peerj.12602/supp-3 Supplemental Information 3 Search of relevant literature containing machine algorithms. 14 relevant literatures including machine algorithms were retrieved

## Additional Information and Declarations

### Data Availability

The following information was supplied regarding data availability: The 10 years of experimental data, including yield and meteorological data, the code for submitting the random forest prediction algorithm, and the literature on machine algorithms retrieved are available in the Supplemental Files.
