# Supplementary figures and images for: Effects of fallow tillage on winter wheat yield and predictions under different precipitation types
Source: PeerJ. 2021 Dec 8;9:e12602. doi: 10.7717/peerj.12602 (PMC8667742; doi:10.7717/peerj.12602)

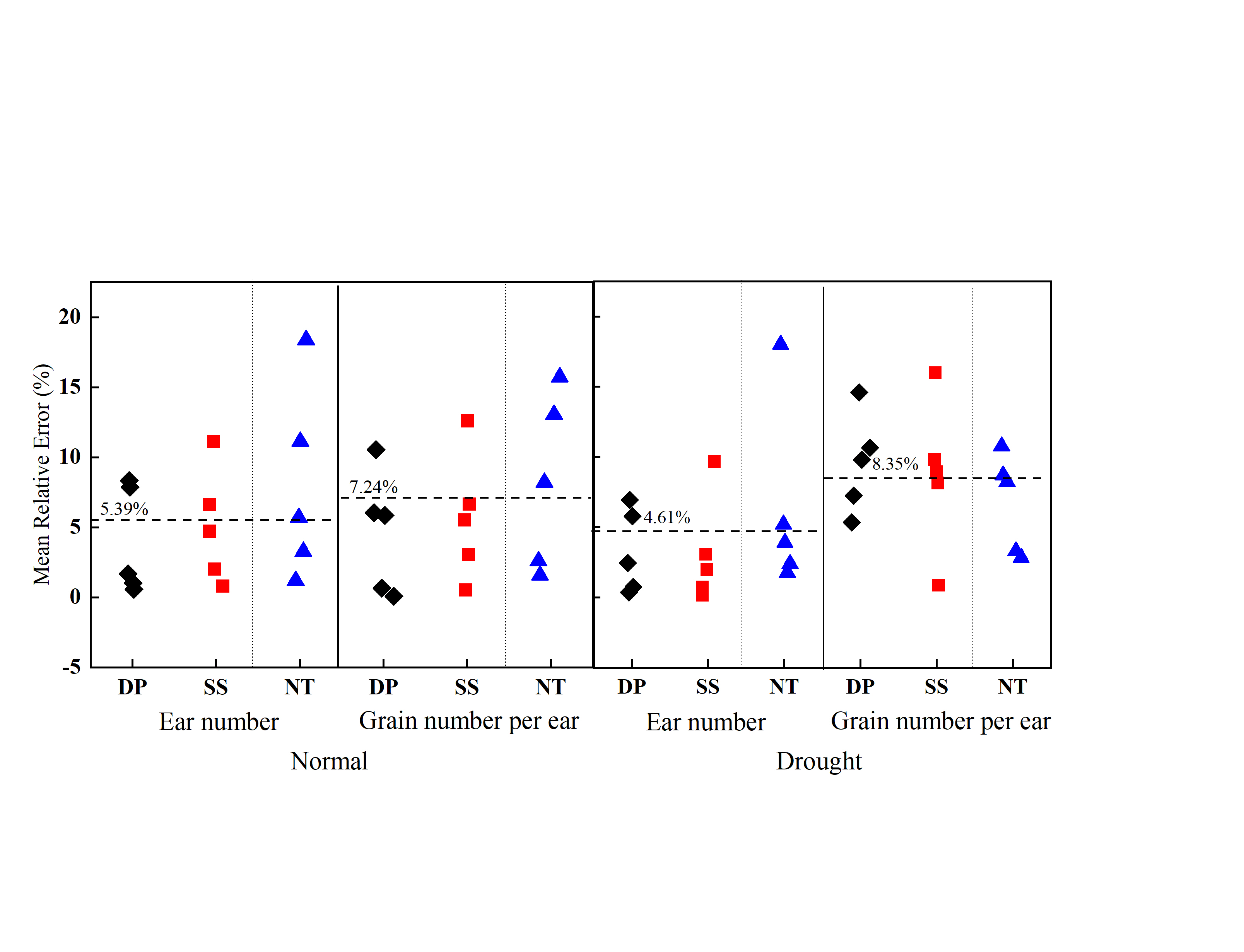

Supplement: Supplemental Information 2 — The result graph and code predicted by the Random Forest algorithm appear in Supplement 2 [file peerj-09-12602-s002.zip › supplemental 2/The predicted results of ear number and grain number per ear/Prediction error of ear number and grain number per ear.jpg]

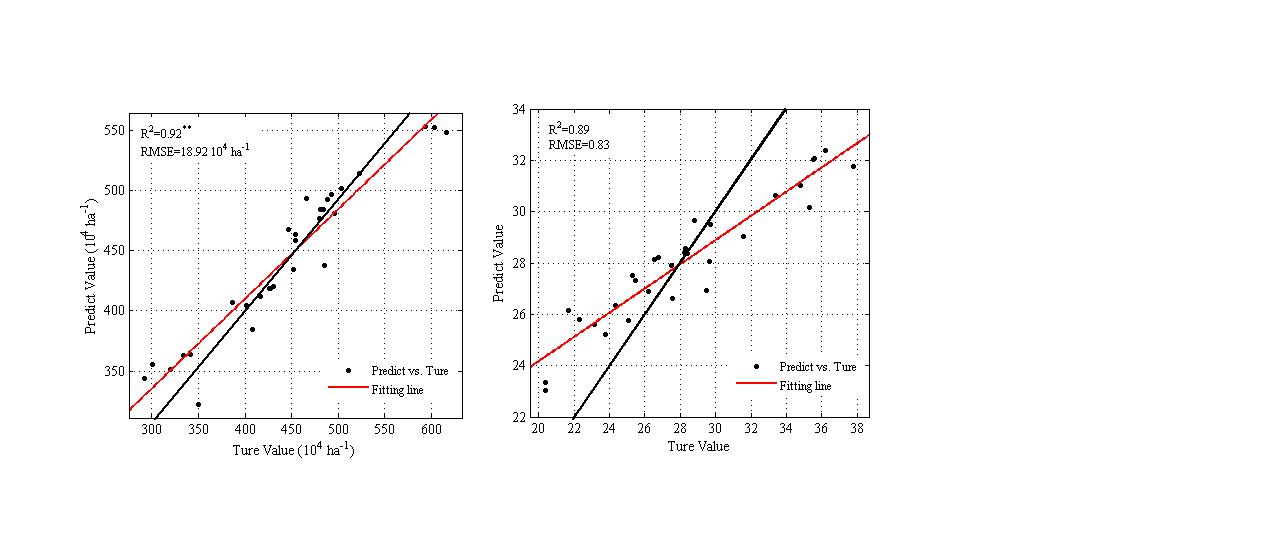

Supplement: Supplemental Information 2 — The result graph and code predicted by the Random Forest algorithm appear in Supplement 2 [file peerj-09-12602-s002.zip › supplemental 2/The predicted results of ear number and grain number per ear/The predicting results of ear number and grain number per ear.jpg]

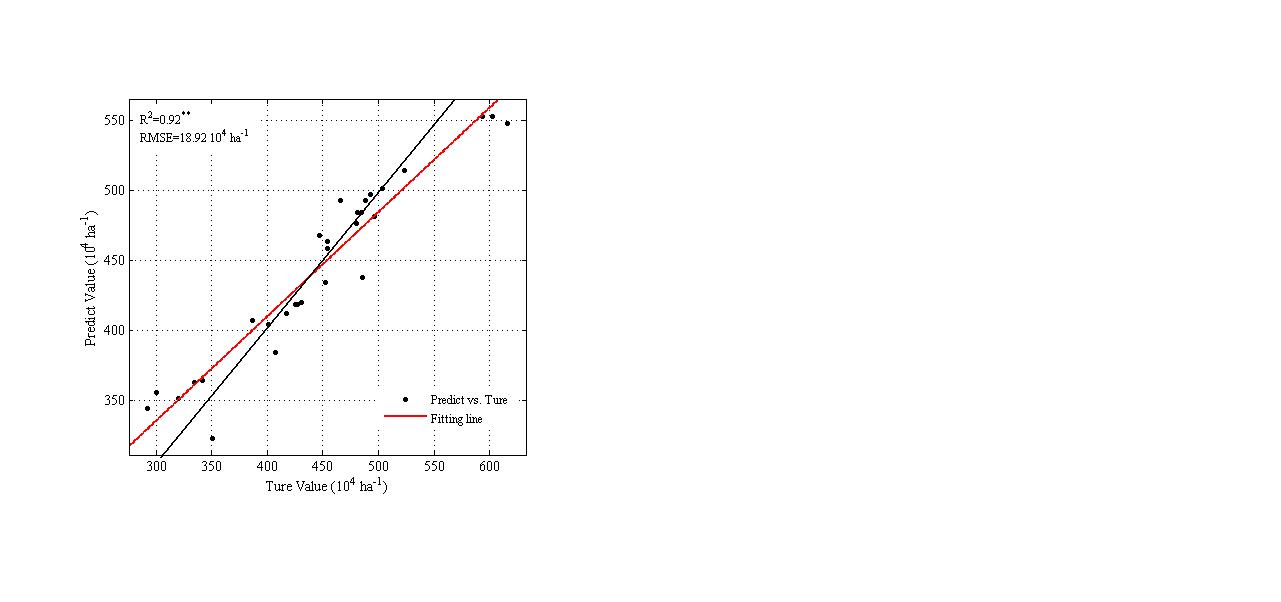

Supplement: Supplemental Information 2 — The result graph and code predicted by the Random Forest algorithm appear in Supplement 2 [file peerj-09-12602-s002.zip › supplemental 2/The predicted results of ear number and grain number per ear/ear number.jpg]

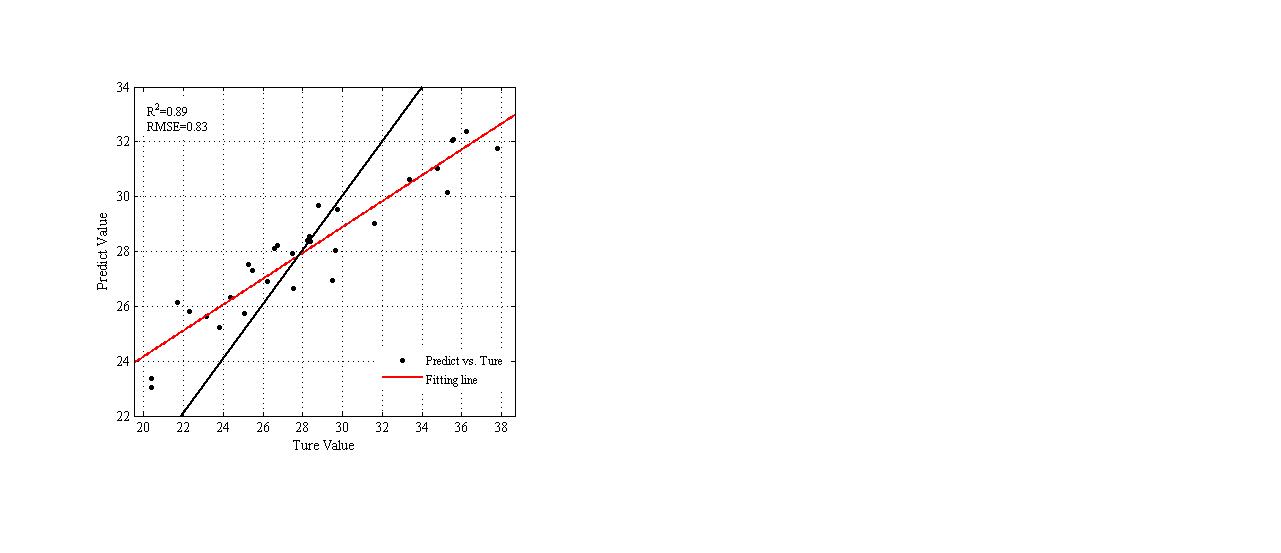

Supplement: Supplemental Information 2 — The result graph and code predicted by the Random Forest algorithm appear in Supplement 2 [file peerj-09-12602-s002.zip › supplemental 2/The predicted results of ear number and grain number per ear/grain number per ear.jpg]

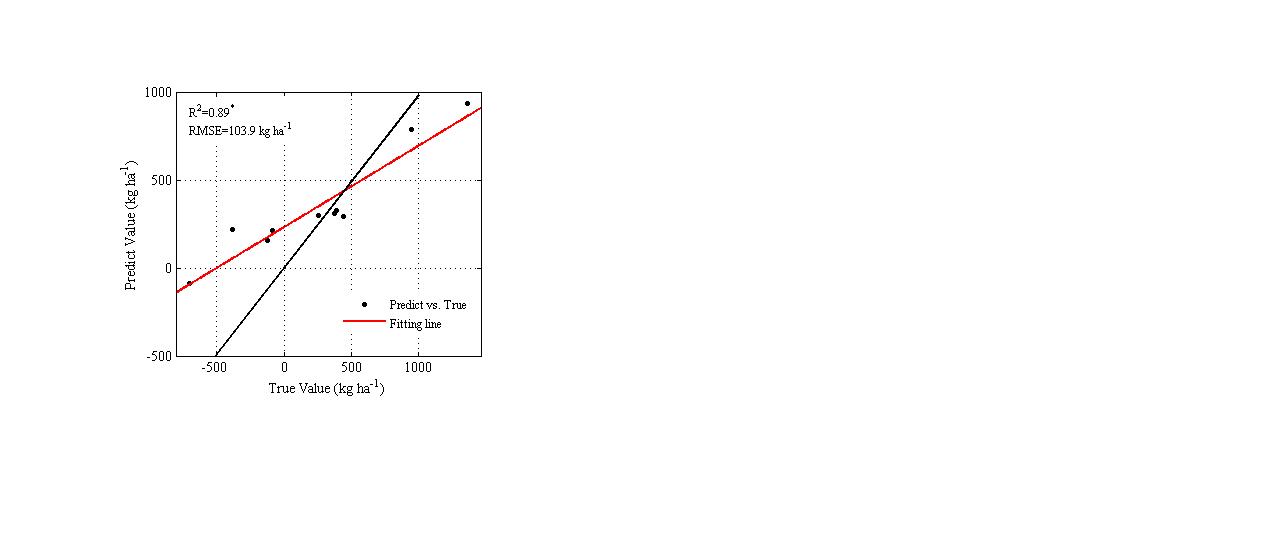

Supplement: Supplemental Information 2 — The result graph and code predicted by the Random Forest algorithm appear in Supplement 2 [file peerj-09-12602-s002.zip › supplemental 2/meteorological yield forecast results/CK Meteorological yield model.jpg]

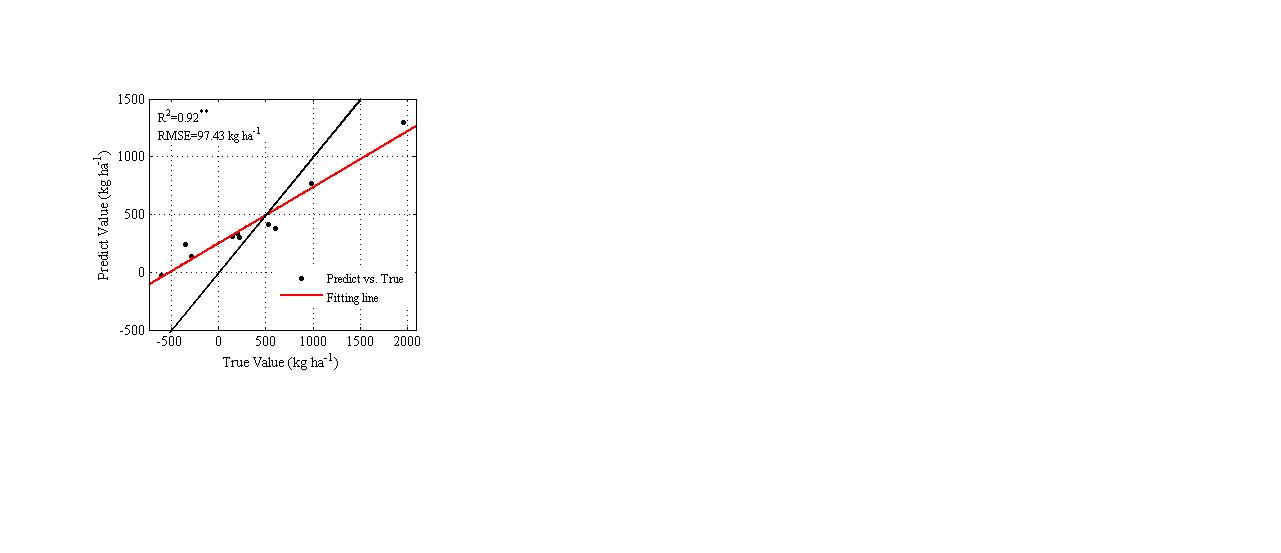

Supplement: Supplemental Information 2 — The result graph and code predicted by the Random Forest algorithm appear in Supplement 2 [file peerj-09-12602-s002.zip › supplemental 2/meteorological yield forecast results/DS Meteorological yield model.jpg]

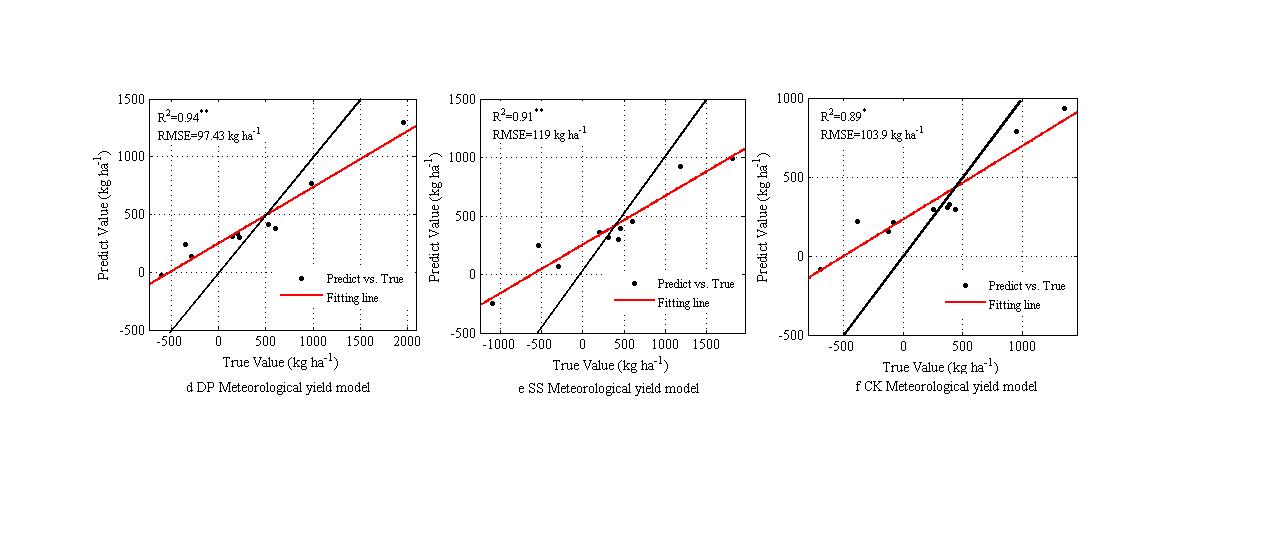

Supplement: Supplemental Information 2 — The result graph and code predicted by the Random Forest algorithm appear in Supplement 2 [file peerj-09-12602-s002.zip › supplemental 2/meteorological yield forecast results/Meteorological yield model.jpg]

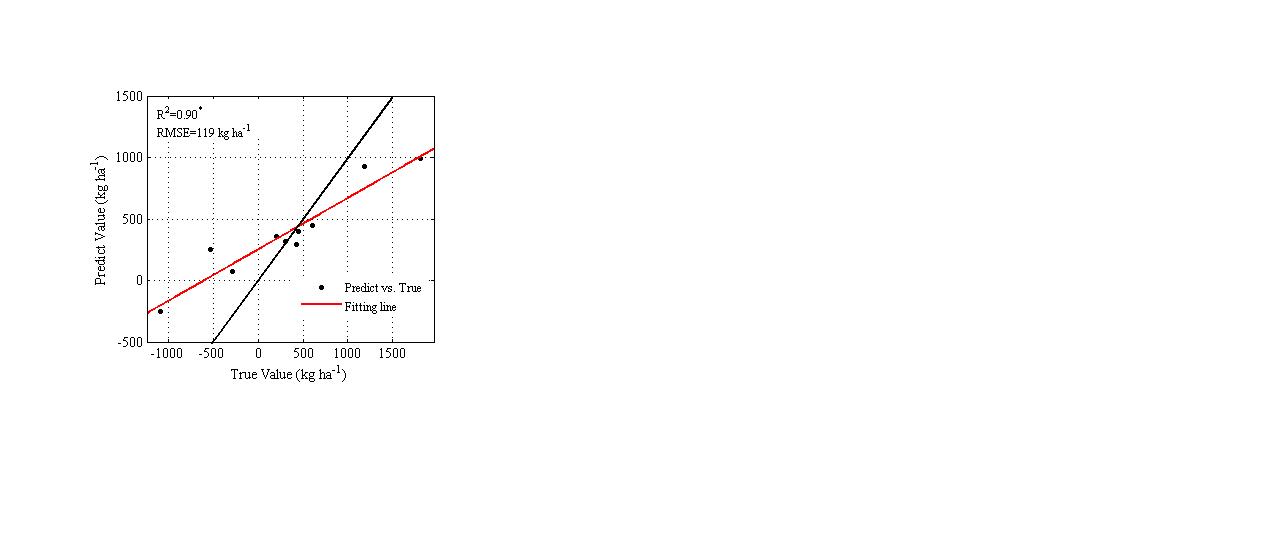

Supplement: Supplemental Information 2 — The result graph and code predicted by the Random Forest algorithm appear in Supplement 2 [file peerj-09-12602-s002.zip › supplemental 2/meteorological yield forecast results/SS Meteorological yield model.jpg]

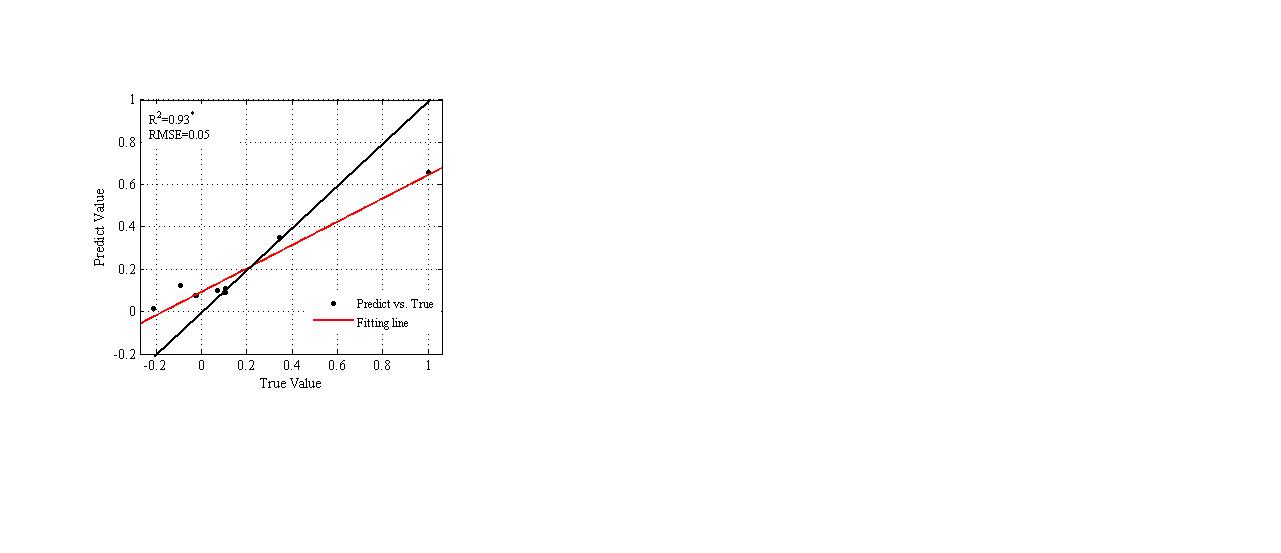

Supplement: Supplemental Information 2 — The result graph and code predicted by the Random Forest algorithm appear in Supplement 2 [file peerj-09-12602-s002.zip › supplemental 2/relative meteorological yield forecast results/CK Relative meteorological yield model.jpg]

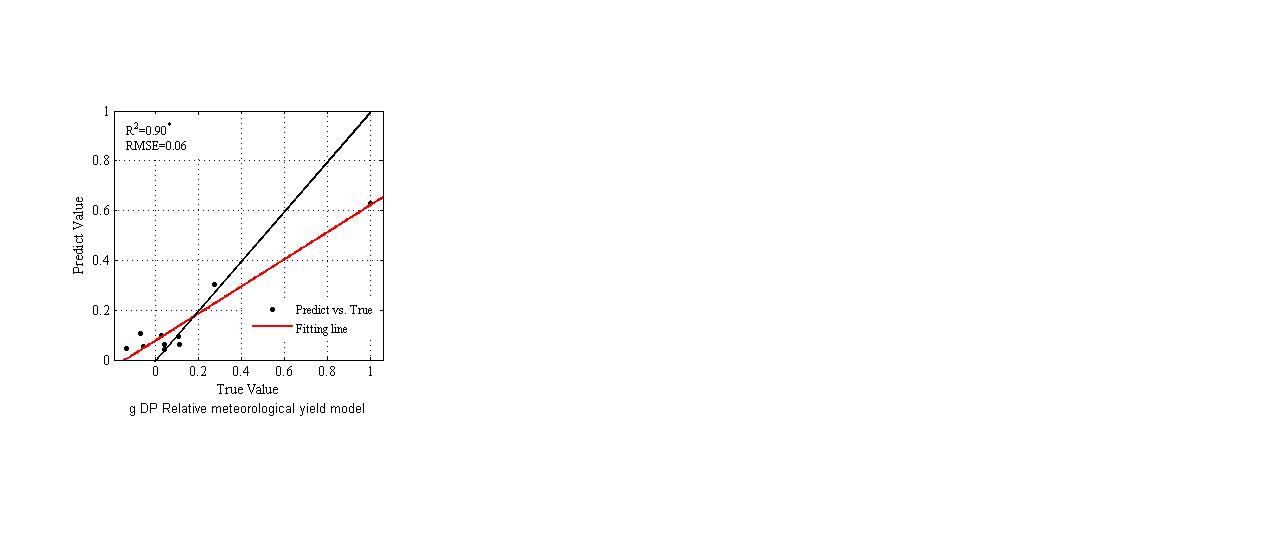

Supplement: Supplemental Information 2 — The result graph and code predicted by the Random Forest algorithm appear in Supplement 2 [file peerj-09-12602-s002.zip › supplemental 2/relative meteorological yield forecast results/DP Relative meteorological yield model.jpg]

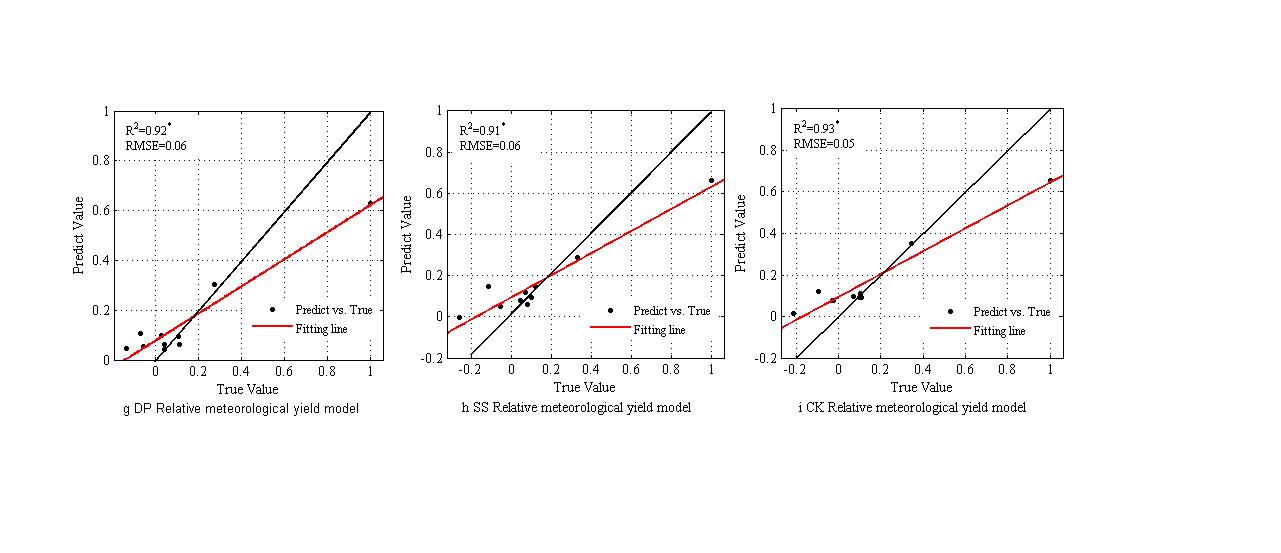

Supplement: Supplemental Information 2 — The result graph and code predicted by the Random Forest algorithm appear in Supplement 2 [file peerj-09-12602-s002.zip › supplemental 2/relative meteorological yield forecast results/Relative meteorological yield model.jpg]

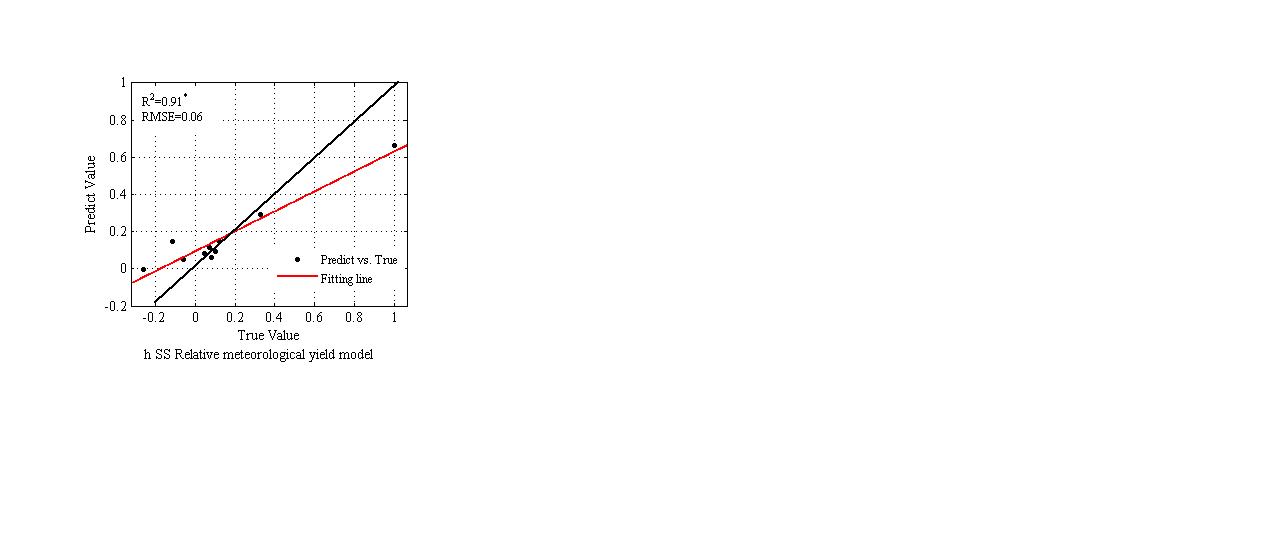

Supplement: Supplemental Information 2 — The result graph and code predicted by the Random Forest algorithm appear in Supplement 2 [file peerj-09-12602-s002.zip › supplemental 2/relative meteorological yield forecast results/SS Relative meteorological yield model.jpg]

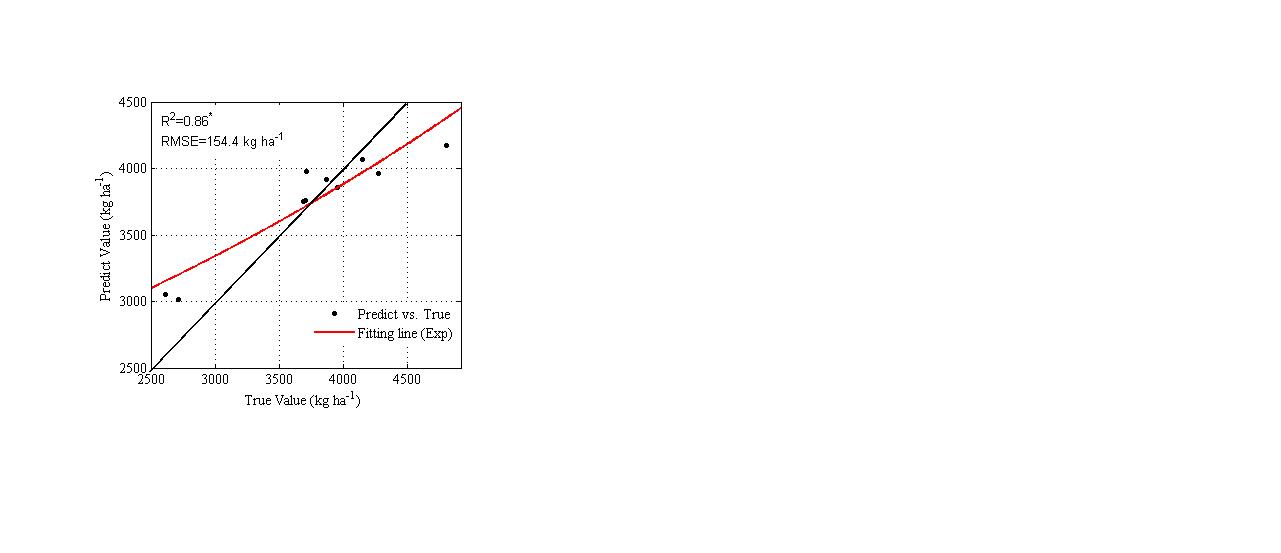

Supplement: Supplemental Information 2 — The result graph and code predicted by the Random Forest algorithm appear in Supplement 2 [file peerj-09-12602-s002.zip › supplemental 2/unit yield forecast results/CK Yield predict.jpg]

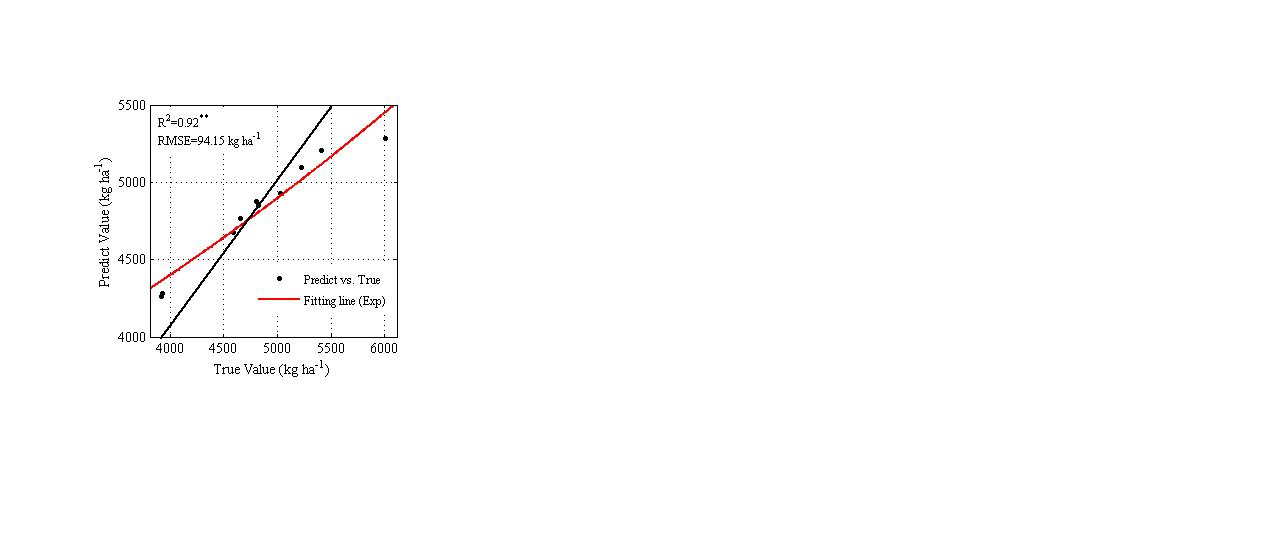

Supplement: Supplemental Information 2 — The result graph and code predicted by the Random Forest algorithm appear in Supplement 2 [file peerj-09-12602-s002.zip › supplemental 2/unit yield forecast results/DP Yield predict.jpg]

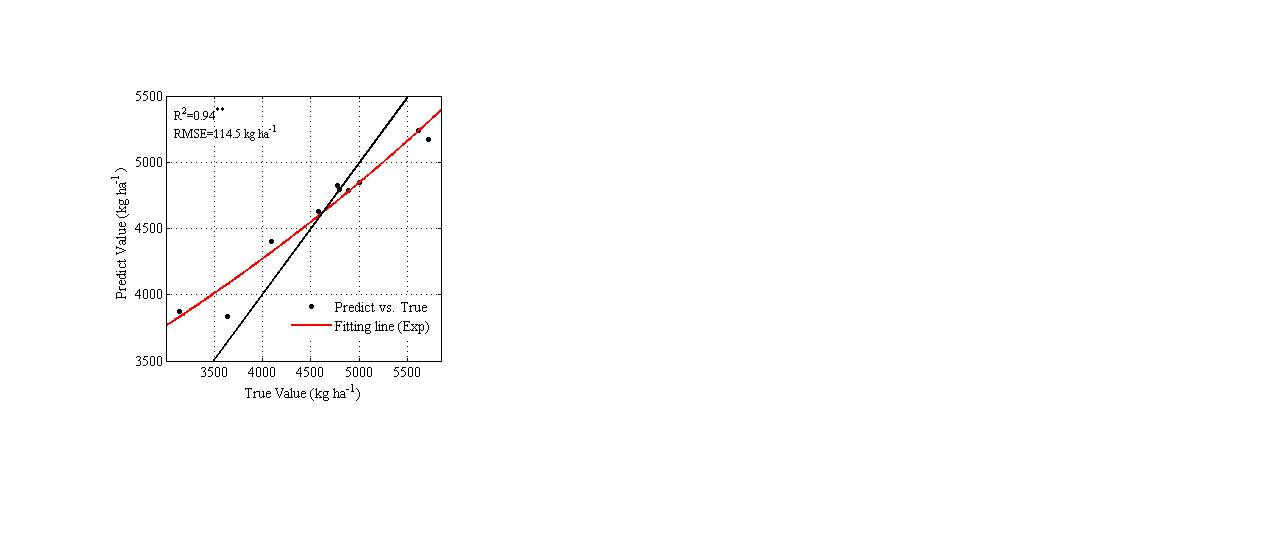

Supplement: Supplemental Information 2 — The result graph and code predicted by the Random Forest algorithm appear in Supplement 2 [file peerj-09-12602-s002.zip › supplemental 2/unit yield forecast results/SS Yield predict.jpg]

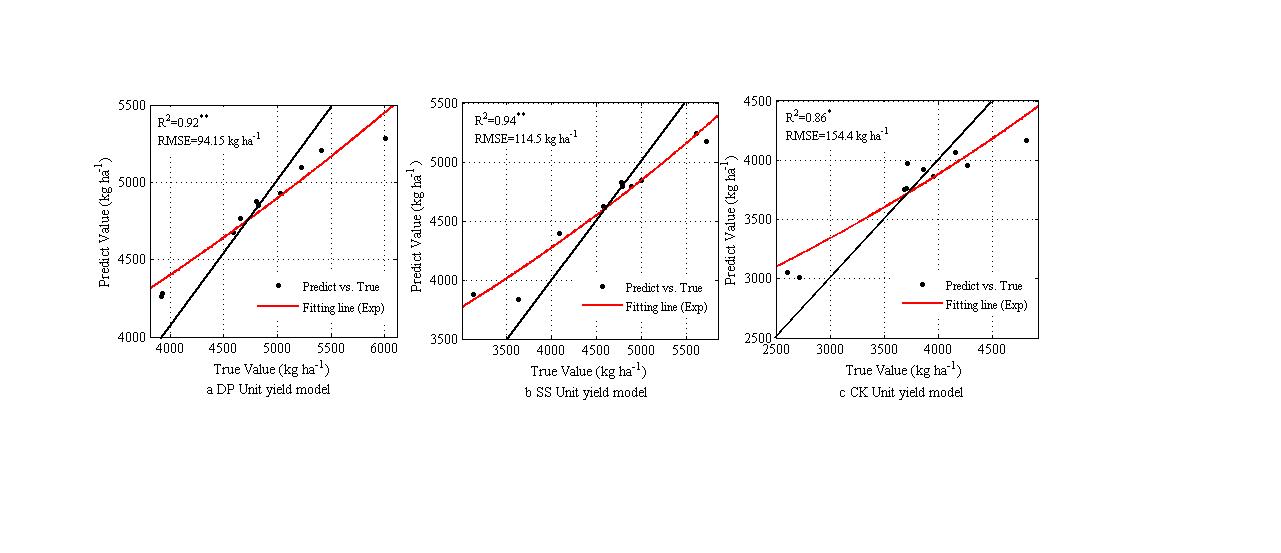

Supplement: Supplemental Information 2 — The result graph and code predicted by the Random Forest algorithm appear in Supplement 2 [file peerj-09-12602-s002.zip › supplemental 2/unit yield forecast results/Yield predict.jpg]
