# Supplementary material for: Effects of fallow tillage on winter wheat yield and predictions under different precipitation types
Source: PeerJ. 2021 Dec 8;9:e12602. doi: 10.7717/peerj.12602 (PMC8667742; doi:10.7717/peerj.12602)
Supplement: Supplemental Information 3 — 14 relevant literatures including machine algorithms were retrieved [file peerj-09-12602-s003.docx]

Supplement 3

References involved are:

**1 Alberto GS, Juan FS, Waldo OB. 2014.** Attribute selection impact on linear and nonlinear regression models for crop yield prediction. *The Scientific World Journal* 509429 DOI 10.1155/2014/509429

**Machine algorithm:** Multiple and stepwise linear regression, Regression Trees, Artificial Neural Networks

**2 Chlingaryan A, Sukkarieh S, Whelan B. 2018.** Machine learning approaches for crop yield prediction and nitrogen status estimation in precision agriculture: a review. *Computers and Electronics in Agriculture* **151**:61-69 DOI 10.1016/j.compag.2018.05.012

**3 Eka B, Fw A, Zh A. 2020.** Estimating wheat yields in Australia using climate records, satellite image time series and machine learning methods-ScienceDirect. *ISPRS Journal of Photogrammetry and Remote Sensing* **160**,124-135 DOI 10.1016/j.isprsjprs.2019.11.008

**Machine algorithm:** Random Forest, Cubist, XgBoost, Multi-Layer Perceptron, Support Vector Regression-Linear, Support Vector Regression-radial basis function, Gaussian Process Regression, K-nearest Neighbours, Multivariate Adaptive Regression Splines

**4 Gopal P, Bhargavi R. 2019.** A novel approach for efficient crop yield prediction. *Computers and Electronics in Agriculture* **165** DOI 104968 DOI 10.1016/j.compag.2019.104968

**Machine algorithm:** Artificial Neural Network, Support Vector Regression, K-Nearest Neighbours and Random Forest

**5 Han JC, Zhang Z, Cao J, Luo YC, Zhang LG, Li ZY, Zhang J. 2020.**Prediction of Winter Wheat Yield Based on Multi-Source Data and Machine Learning in China. *Remote Sensing* ***12***, 236 DOI 10.3390/rs12020236

**Machine algorithm:** Support vector machine (SVM), Gaussian process regression (GPR), and random forest (RF)

**6 Janmejay Pant, R.P. Pant, Manoj Kumar Singh, Devesh Pratap Singh, Himanshu Pant. 2021.** Analysis of agricultural crop yield prediction using statistical techniques of machine learning. *Materials Today: Proceedings* DOI 10.1016/j.matpr.2021.01.948

**Machine algorithm:** Gradient Boosting Regressor, Random Forest Regressor, SVM, Decision Tree Regressor

**7 Jeong JH, Resop JP, Mueller ND, Fleisher DH, Yun K, Butler EE, Dennis J, Timlin, Kyo-Moon S, James SG, Vangimalla Rr, Soo-Hyung K.** 2016. Random Forests for Global and Regional Crop Yield Predictions. *PLoS ONE* **11(6)**: e0156571 DOI 10.1371/journal.pone.0156571

**Machine algorithm:** Random Forest

**8 Kanwal HH, Ahmad I, Ahmad A, Yongfu L. 2021.** Yield forecasting and assessment of interannual wheat yield variability using machine learning approach semiarid environment. *Pakistan Journal of agricultural sciences* **58(2)**:461-470 DOI 10.21162/PAKJAS/21.661

**Machine algorithm:** Linear Discriminant Analysis (LDA), Quadratic Discriminant Analysis (QDA), K-Nearest Neighbor (KNN), Support Vector Machine (SVM) with linear kernel, SVM with Radial Basis Kernel, Decision Trees, Boosting, and Random Forests

**9 Krause M, Crossman S, Dumond Todd, Lott Rodman, Swede J, Arliss Scott, Robbins R, Ochs Daniel, Gore Michael. 2020.** Random forest regression for optimizing variable planting rates for corn and soybean using topographical and soil data. *Agronomy Journal* **112**:5045-5066 DOI 10.1002/agj2.20442

**Machine algorithm:** Random Forest

**10 Maya Gopal P S; Bhargavi R. 2019.** Performance evaluation of best feature subsets for crop yield prediction using machine learning algorithms. *Applied Artificial Intelligence* **33:7**, 621-642 DOI: 10.1080/08839514.2019.1592343

**Machine algorithm:** Artificial Neural Network, Support Vector Regression, K-Nearest Neighbour and Random Forest (RF)

**11 Ramos A, Osco LP, Furuya D, Gonalves WN, Pistori H. 2020.** A random forest ranking approach to predict yield in maize with uav-based vegetation spectral indices. *Computers and Electronics in Agriculture* **178**, 105791 DOI 10.1016/j.compag.2020.105791

**Machine algorithm:** Support vector machine, artificial neural network, Random Forest

**12 Sharifi A. 2020.** Yield prediction with machine learning algorithms and satellite images. *Journal of the Science of Food and Agriculture* **101(3),**891-896 DOI 10.1002/jsfa.10696

**Machine algorithm:** Backpropagation neural network, Decision tree, K-nearest neighbor regression, the Gaussian Process Regression algorithm

**13 Shook J, Gangopadhyay T, Wu L, Ganapathysubramanian B, Sarkar S, Singh AK. 2021.** Crop yield prediction integrating genotype and weather variables using deep learning. *PLoS ONE* **16(6):**e0252402 DOI 10.1371/journal.pone.0252402

**Machine algorithm:** Random Forest

**14 Li L, Wang B, Feng P, Wang H, Yu Q. 2021.** Crop yield forecasting and associated optimum lead time analysis based on multi-source environmental data across China. *Agricultural and Forest Meteorology* **308-309(4):** 108558 DOI 10.1016/j.agrformet.2021.108558

**Machine algorithm:** Random Forest
